# Social Coordination Maintained by Progressive Delay of Coordination-Dependent Reinforcement

**DOI:** 10.3390/bs16060967

**Published:** 2026-06-11

**Authors:** Firdavs Khaydarov, Kennon A. Lattal

**Affiliations:** 1 Department of Psychology, Southeastern Louisiana University, Hammond, LA 70402, USA; 2 Department of Psychology, West Virginia University, Morgantown, WV 26506, USA; andy.lattal@mail.wvu.edu

**Keywords:** social coordination, coordination-dependent reinforcement, delay of reinforcement, progressive delay, signaled delay, unsignaled delay, pigeons

## Abstract

Social coordination refers to conjoint responses of two or more organisms that produce consequences for each and can be maintained by coordination-dependent reinforcement. Previous experimental investigations of coordination typically arranged immediate reinforcement following a coordinated response, leaving the effects of delayed reinforcement on coordination largely unexplored. The present investigation examined the effects of delayed reinforcement on coordinated responding across two experiments using pairs of pigeons. Experiment 1 evaluated the effects of progressively increasing delays of reinforcement on coordinated responding and assessed whether coordination-reinforcer dependency influenced the persistence of coordination. Coordination ratios and coordination rates generally were lower during delayed relative to immediate reinforcement. In addition, break points, which were used as a measure of persistence, were consistently higher during coordination-dependent than coordination-independent delayed reinforcement. Experiment 2 compared coordinated responding maintained under signaled and unsignaled progressively increasing delays of coordination-dependent reinforcement. Coordination generally persisted at higher levels during signaled than unsignaled delays, and coordination ratios and coordination rates maintained under signaled delays more closely resembled performance maintained under immediate reinforcement. These findings suggest that delayed reinforcement weakens coordinated responding and that delay-correlated stimuli may attenuate some of the disruptive effects of delay on coordinated behavior.

## 1. Introduction

Social coordination (hereafter used interchangeably with coordination and coordinated responding) refers to conjoint responses of two or more organisms with respect to a common environment that produce response-maintaining consequences for each organism. Such coordinated behavior often produces consequences that would be difficult or impossible to obtain through the behavior of a single organism. Observations of coordinated hunting in chimpanzees, for example, illustrate how individuals assume complementary roles that increase the likelihood of capturing prey (Boesch, 2002). Once prey is captured, resources are shared among participants, suggesting that coordinated behavior may be sustained by environmental consequences that follow the joint actions of multiple individuals (Stanford et al., 1994).

From a behavior-analytic perspective, social coordination can be understood as an operant behavior maintained by reinforcement contingencies that depend on the responding of multiple individuals rather than that of a single organism (Skinner, 1953). Lindsley (1963) described such arrangements as mutual reinforcement (hereafter referred to as coordination-dependent reinforcement), in which coordinated responding produces reinforcement for each participant. If coordinated responding is to be regarded as an operant, however, it must demonstrate the defining characteristics of operant behavior.

A defining characteristic of operant behavior is that it occurs differentially in the presence versus absence of a contingency between responding and its consequences. Experimentally, such differential control may be demonstrated by showing that a response is maintained differentially by the presence and absence of a response–reinforcer dependency and is sensitive to changes in reinforcement conditions (Catania, 1973).

Consistent with these defining characteristics of operant behavior, several studies have demonstrated operant properties of coordinated responding. Tan and Hackenberg (2016), for instance, compared coordinated responding under coordination-dependent reinforcement with yoked control conditions in which reinforcement occurred independently of coordination. Their results showed that coordination was substantially higher when reinforcement depended on coordinated responding, suggesting that coordination is sensitive to the contingency between coordinated behavior and reinforcement.

In addition to demonstrating that coordinated responding is influenced by coordination–reinforcer dependency, research has shown that coordination also is sensitive to schedules of reinforcement. Specifically, de Carvalho et al. (2018) examined coordination under fixed-ratio (FR) 1, FR 10, and variable-ratio (VR) 10 schedules of coordination-dependent reinforcement and found that these arrangements produced distinct rates and patterns of coordination. Coordination, for instance, was highest under the VR 10 schedule relative to the FR 1 and FR 10 schedules. Conversely, coordination was higher under the FR 10 schedule than under the FR 1 schedule. In addition, coordination postreinforcement pauses (PRPs), defined as the time from the end of reinforcer delivery to the first coordinated response, were longer under the FR 10 schedule than under either the VR 10 or FR 1 schedules. Extending these findings to interval schedules, Todorov et al. (2020) compared fixed-interval (FI) and variable-interval (VI) schedules of coordination-dependent reinforcement and likewise found differential effects on coordinated responding, with higher rates of coordination occurring during VI schedules than during FI schedules.

In the aforementioned investigations (de Carvalho et al., 2018; Tan & Hackenberg, 2016; Todorov et al., 2020), coordination was examined primarily through manipulations of consequence events. Operant behavior, however, also may come under the control of antecedent events. When responding occurs differentially across stimulus conditions because reinforcement has been correlated with responding in the presence of one stimulus but not another, the response is said to be a discriminated operant (Catania, 1973). Consistent with this property of discriminated operant behavior, coordination also has been shown to come under discriminative control. Lattal and Okouchi (2023), for example, differentially reinforced coordinated responding in the presence of one stimulus but not another, resulting in higher levels of coordination in the presence of the stimulus correlated with coordination-dependent reinforcement.

Beyond demonstrating sensitivity to response–reinforcer contingencies, schedules of reinforcement, and discriminative control, operant behavior also is characterized by differential sensitivity to other parameters of reinforcement such as frequency, magnitude, and delay. de Carvalho et al. (2020), for instance, examined coordination by parametrically manipulating the response requirement of a coordination-dependent FR schedule, thereby also systematically manipulating reinforcement frequency. This resulted in an inverted U-shaped function between reinforcement rates and coordination rates, which was attributed to the interaction between ratio size requirement and reinforcement frequency (cf. dos Santos et al., 2023; Killeen, 1969). In most experimental analyses of coordination, however, reinforcement has been delivered immediately following the coordinated response (e.g., de Carvalho et al., 2018, 2020, 2019; Tan & Hackenberg, 2016; Todorov et al., 2020), thereby preserving temporal contiguity between responding and its consequences. In natural environments, however, consequences of coordinated responses are often delayed from the behaviors that produce them. For example, members of a surgical team must coordinate their behavior to achieve successful patient outcomes, although reinforcement for such coordination may be delayed until completion of the procedure. Likewise, collaborative workplace projects often involve coordinated contributions by multiple individuals, with consequences such as financial compensation, social recognition, or project completion occurring only after substantial delays. These examples illustrate that delayed consequences are a common feature of socially coordinated human behavior. Although coordinated behavior in such settings may ultimately produce important outcomes, the temporal separation between responding and reinforcement may weaken the maintenance of coordinated responding. To date, however, no investigations, to the authors’ knowledge, have examined the effects of delayed reinforcement on coordinated responding.

In analyses of individual operants, response rates have been shown to decrease systematically as a function of increasing delay duration (e.g., Ferster, 1953; Reilly & Lattal, 2004; Richards, 1981; Sizemore & Lattal, 1978). One variable that has been shown to attenuate the response-reducing effects of delayed reinforcement is the presence of a stimulus correlated with the delay period. When a stimulus change accompanies the delay between a response and reinforcer delivery, responding is often maintained at higher levels compared to conditions in which the delay occurs without such a signal (e.g., Ferster, 1953; Lattal, 1984; Richards, 1981; Schaal & Branch, 1988). Signals correlated with the delay period, therefore, may attenuate some of the disruptive effects produced by temporal separation between responses and their consequences. Although these effects have been demonstrated extensively with individual operant responses, it remains unclear whether signaled and unsignaled delays similarly affect the maintenance of coordinated behavior under coordination-dependent reinforcement.

The purpose of the present investigation, therefore, was to evaluate (1) the occurrence of coordinated responding across progressively delayed reinforcement and (2) the effects of delay-correlated stimuli on the persistence of coordinated responding. Delay duration was arranged using a progressive delay of coordination-dependent reinforcement procedure, in which delays increased systematically across a session. A progressive delay procedure was used because it permitted assessment of coordination across a broad range of delay values within each session and across a relatively limited number of sessions. In addition, this arrangement reduced the need for prolonged exposure to multiple fixed-delay conditions that would have been required in more traditional sequential or alternating-baseline procedures. Such procedures may increase the likelihood of uncontrolled behavioral changes associated with extended experimental histories, a concern commonly noted in single-subject research requiring prolonged exposure to successive conditions (Kazdin, 2011; Sidman, 1960). Finally, the progressive-delay arrangement minimized differences in reinforcement frequency and distribution that often accompany comparisons between separate immediate- and delayed-reinforcement conditions (cf. Reilly & Lattal, 2004). To isolate the effects of delayed reinforcement from changes in reinforcement frequency that accompany increasing delay durations, a yoked-interval schedule of coordination-dependent reinforcement was included as a point of comparison. In addition, to isolate the effects of coordination-dependent reinforcement contingencies, a control condition consisting of progressive-delay and yoked-interval schedules of coordination-independent reinforcement (i.e., with each member of the dyad exposed to separate and independent reinforcement contingencies) was included.

## 2. Experiment 1

The purpose of Experiment 1 was to examine the effects of delayed reinforcement on coordinated responding. Two comparisons were of primary interest. First, coordinated responding maintained by delayed reinforcement was compared to that maintained by immediate reinforcement under coordination-dependent reinforcement contingencies to evaluate the effects of delayed reinforcement on coordinated responding. This comparison permitted an assessment of whether progressively increasing delays systematically weakened the maintenance of coordinated responding relative to conditions in which reinforcement was delivered immediately. Second, the effects of coordination-dependent delayed reinforcement were compared to those of coordination-independent delayed reinforcement to evaluate the role of coordination–reinforcer dependency in the persistence of coordinated responding when reinforcement was delayed. Previous investigations examining the role of coordination–reinforcer dependency in the acquisition and maintenance of coordinated responding primarily have focused on measures such as coordination ratios and coordination rates (e.g., de Carvalho et al., 2018; Tan & Hackenberg, 2016; Todorov et al., 2020). Another approach to the analysis of operant behavior, however, is to examine its persistence under conditions that disrupt responding (cf. Nevin, 1974). In studies employing progressive-ratio schedules, persistence of behavior has commonly been assessed using a break point, typically defined as the largest ratio requirement completed before responding ceases for a specified period of time (e.g., Hodos, 1961; Stafford & Branch, 1998). In the present investigation, persistence of coordination was indexed by an analogous break-point measure, defined as the maximum delay value at which coordinated responding was maintained. Because persistence of coordinated responding was the primary dependent measure of interest in the comparison between coordination-dependent and coordination-independent delayed reinforcement, reinforcement rates were not equated across these conditions. Accordingly, these arrangements were examined separately.

### 2.1. Method

#### 2.1.1. Subjects

Eight mature male White Carneau pigeons were maintained at approximately 80% (±40 g) of their free-feeding weights. Post-session feeding was provided as required to maintain the target weight. The pigeons were housed in separate cages with a 12:12 h light/dark cycle in the vivarium. Continuous water was available in their home cages. Each pigeon had a history of keypecking on different schedules of reinforcement. All procedures conformed to the *Guide for the Care and Use of Laboratory Animals* (National Research Council, 2011) and were approved by the Institutional Animal Care and Use Committee at West Virginia University.

#### 2.1.2. Apparatus

**Figure 1:**
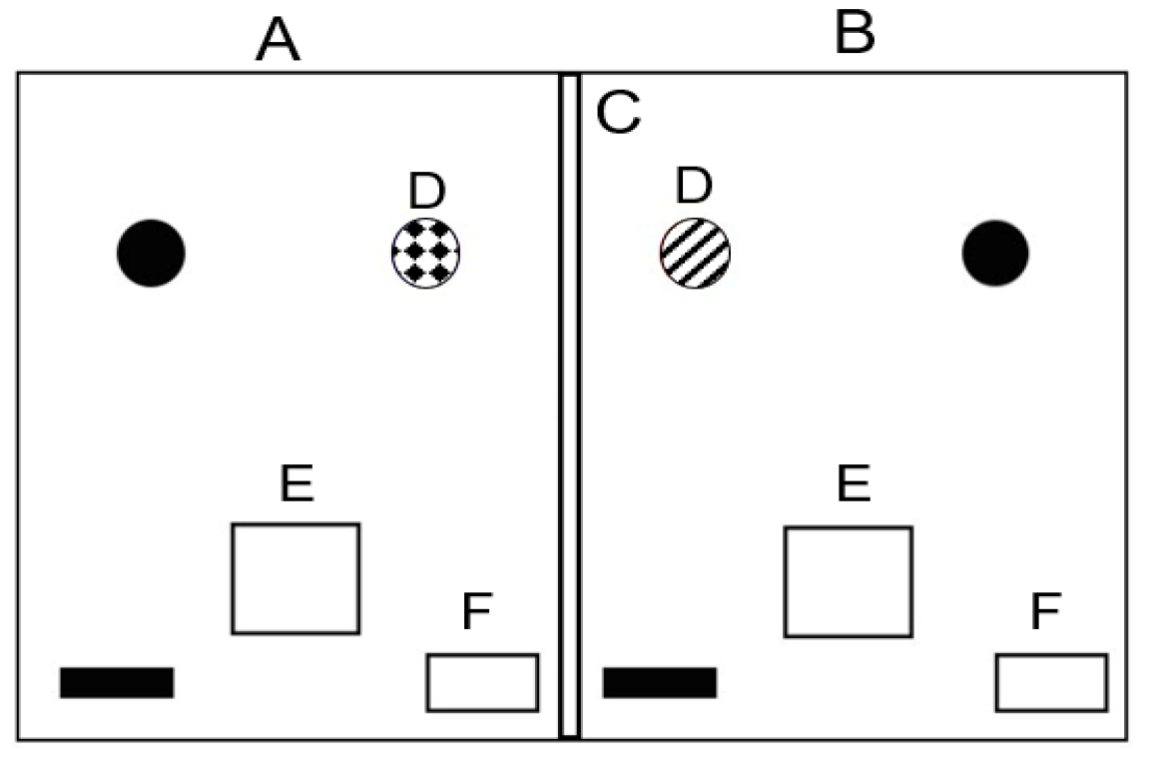
Diagram of the operant chamber used in the experiments. The drawing is not to scale. The chamber was divided into two equal-sized compartments (A and B) by a central transparent plastic partition (C). The active response keys (D) in each compartment were adjacent to either side of the plastic partition. Each work panel also contained a feeder aperture through which reinforcers were delivered (E) and a houselight (F) for general illumination. Solid black circles indicate inactive response keys that were present but not used in the experiment, and solid black rectangles indicate sound generators that were present but not used.

An operant chamber housing two separate work panels was used. The plywood chamber was 70 cm wide, 40 cm deep, and 60 cm long and is shown in Figure 1. Two aluminum work panels (A and B) were adjacent to one another, separated by a transparent plastic partition. Each panel contained two 2-cm diameter Ralph Gerbrands Company response keys. Only the right key of Panel A and the left key of Panel B, which were adjacent to one another, were used. The centers of the active keys were located approximately 10 cm on either side of the transparent plastic partition and 10 cm from the center of each to the chamber ceiling. Each key was transilluminated by different colored 28 V DC lamps, as described below, and required a force of approximately 0.15 N to operate. Reinforcement was 3 s access to grain from a Ralph Gerbrands Company hopper located behind a 4.5 cm square feeder aperture located on the midline of the work panels 10 cm from the floor. During reinforcement, the aperture was illuminated by a white light. General chamber illumination was provided by two houselights located in the lower right corner of each work panel throughout the session except during reinforcement. White noise masked extraneous sounds. An IBM-compatible desktop computer operating MED-PC 7 software controlled the experiment from a separate room.

#### 2.1.3. Experimental Design

Coordinated responding was examined using two separate two-component multiple schedules, the components of which alternated daily (cf. Bloomfield, 1967). One was a Coordination-Dependent Multiple Schedule and consisted of a progressive-delay component (Component 1) followed by a yoked-immediate-reinforcement component (Component 2). The second was a Coordination-Independent Multiple Schedule and likewise consisted of a progressive-delay component (Component 1) followed by a yoked-immediate-reinforcement component (Component 2).

Within each multiple schedule, dyads were exposed to the progressive-delay component on one day and to the corresponding yoked-immediate-reinforcement component on the following day. The progressive-delay component always preceded the yoked component because the obtained interreinforcer intervals (IRIs) generated during the progressive-delay session were reproduced during the subsequent yoked-immediate-reinforcement session.

The comparison between delayed and yoked-immediate-reinforcement components within the Coordination-Dependent Multiple Schedule permitted an assessment of the effects of progressively increasing delays while controlling for changes in reinforcement frequency and distribution across the session. The comparison between the progressive-delay components of the Coordination-Dependent and Coordination-Independent Multiple Schedules permitted an assessment of whether persistence of coordinated responding was maintained by the coordination contingency rather than emerging as an incidental byproduct of individual responding. If coordinated responding were simply an incidental byproduct of independently reinforced responding, then systematic differences in break points between the two arrangements would not be expected. The order of exposure to the multiple schedules was counterbalanced across dyads, as shown in Table 1.

#### 2.1.4. General Procedure

Eight pigeons were paired randomly to form four dyads. Each member of a dyad was assigned to one of the two compartments of the operant chamber. Both dyad composition and compartment assignment remained fixed throughout the experiment. Sessions were conducted seven days per week unless a subject failed to meet the weight criterion described in the Subjects section above. A 3 min blackout preceded each session to minimize the effects of handling on responding. Following the blackout period, the onset of the houselights and response keys, simultaneously in both compartments, signaled the beginning of the session.

**Figure 2:**
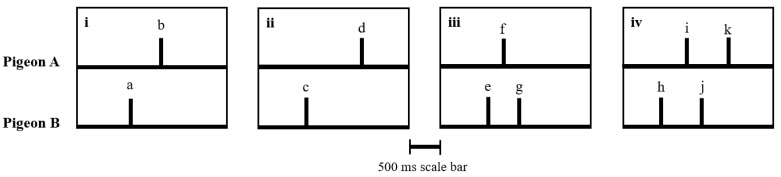
Schematic representation of coordinated and uncoordinated responses under the 500 ms coordination criterion. Example (**i**) illustrates a coordinated response. Example (**ii**) illustrates two responses separated by more than 500 ms and therefore recorded as uncoordinated responses. Example (**iii**) illustrates one coordinated response and one uncoordinated response. Example (**iv**) illustrates two coordinated responses. The scale bar represents a 500 ms interval.

A coordinated response was defined as responses by both pigeons occurring within 500 ms of one another. A 500 ms coordination criterion was selected because previous investigations of coordinated responding have used similar temporal windows and demonstrated reliable acquisition and maintenance of coordination under this criterion (e.g., Skinner, 1962; Tan & Hackenberg, 2016; Todorov et al., 2020). Examples of coordinated and uncoordinated responses are shown in Figure 2. In example (i), responses by Pigeon A and Pigeon B occur within 500 ms of one another and therefore are recorded as a coordinated response. In example (ii), responses (c) and (d) occur more than 500 ms apart and therefore are recorded as uncoordinated responses. In example (iii), two keypecks (e and g) occur within 500 ms of one another by Pigeon B, while only one keypeck (f) by Pigeon A occurs. In this case, keypecks (e) and (f) are counted as a coordinated response, whereas the second keypeck by Pigeon B (g) is counted as an uncoordinated response. In example (iv), two keypecks occur within 500 ms of one another by Pigeon A (i and k) and Pigeon B (h and j). In this case, two coordinated responses (h and i, j and k) are recorded.

#### 2.1.5. Preliminary Training

Training began with an FR 1 schedule of coordination-dependent reinforcement in which every coordinated response produced simultaneous reinforcement for both members of a dyad. The FR requirement subsequently was increased incrementally across sessions until a terminal value of FR 10 was reached. Following FR training, dyads were exposed to a VI schedule of coordination-dependent reinforcement in which the first coordinated response following the elapse of the programmed interval produced simultaneous reinforcement for both pigeons. The mean VI value was increased in 5 s increments every three sessions to a terminal value of VI 30 s. All VI schedules consisted of 10 intervals generated according to the Fleshler and Hoffman (1962) progression. Advancement to the subsequent VI value required a coordination ratio of at least 0.20 across three successive sessions.

Once coordination was maintained under the VI 30 s schedule of coordination-dependent reinforcement, progressively increasing delays were introduced. Preliminary observations indicated substantial variability across dyads in sensitivity to delayed reinforcement. Delay increments, therefore, were selected individually for each dyad to permit evaluation across progressively increasing delays while maintaining coordinated responding throughout the session. Delay increments were determined empirically during pilot sessions by systematically increasing delay values across sessions until fewer than 30 reinforcers were obtained per session. The highest delay increment associated with at least 30 reinforcers per session was then selected for each dyad. Dyads 263–028 and 482–647 experienced delay increments of 0.10 s, whereas Dyads 215–448 and 321–074 experienced delay increments of 0.25 s. Thus, delay increments were selected to ensure that a minimum of 30 reinforcers were obtained per session.

#### 2.1.6. Common Features of the Two Multiple Schedules

##### Progressive-Delay Component

In the progressive-delay component, a tandem VI 20 s (initial link) progressive-time (PT) schedule was in effect. The PT schedule served as the terminal link, hereafter referred to as the delay period. Delay increments were either 0.10 s or 0.25 s, depending on the dyad. No stimulus change accompanied the delay periods, hence the delays were unsignaled. Responses occurring during the delay period were recorded but otherwise were without effect and did not reset or alter the programmed delay duration. The first reinforcer of each session was delivered immediately (PT = 0) after the completion of the first VI interval. After each subsequent reinforcer delivery, the delay period was increased by 0.10 s for Dyads 263–028 and 482–647, whereas the delays for Dyads 215–448 and 321–074 were increased by 0.25 s. For both coordination-dependent and coordination-independent progressive-delay arrangements, sessions terminated following 5 min without a coordinated response.

##### Yoked-Immediate-Reinforcement Component

In the yoked-immediate-reinforcement component, sequences of interreinforcer intervals (IRIs) generated during the preceding session of the progressive-delay component were used in the subsequent session. Specifically, each yoked IRI consisted of the entire obtained interreinforcer interval from the progressive-delay component, including both the obtained VI-link interval and the programmed delay period preceding reinforcement delivery. Sessions were terminated on completion of the entire sequence of yoked IRIs. Because the final IRI of the progressive-delay component was associated with the absence of a coordinated response for 5 min and subsequent session termination without reinforcer delivery, no reinforcer was presented during the final yoked interval. Instead, sessions terminated once the final yoked IRI elapsed independently of further responding.

##### Stability Criteria

Each multiple schedule remained in effect for a minimum of 40 sessions, with each component presented at least 20 times, and until responding in both components concurrently satisfied a stability criterion based on coordination ratios (described in the Measurement section below). Stability was assessed for each component across its final six sessions and was defined as the absence of systematic increasing or decreasing trends in coordination ratios across the six-session block. In addition, the mean coordination ratio of the first three sessions and the mean coordination ratio of the last three sessions each differed by no more than 15% from the overall six-session mean (cf. Schoenfeld et al., 1956).

#### 2.1.7. Coordination-Dependent Multiple Schedule

##### Progressive-Delay Component

A progressive-delay schedule was in effect for both members of a dyad. After the elapse of the VI interval, the first coordinated response initiated the delay period simultaneously for both dyad members. At the end of the delay period, reinforcement was delivered simultaneously to both dyad members independently of further responding.

##### Yoked-Immediate-Reinforcement Component

A single sequence of IRIs generated from coordination-dependent delayed reinforcement was used to create a schedule of coordination-dependent immediate reinforcement. After the elapse of the yoked interval, the first coordinated response resulted in immediate reinforcement delivery to both dyad members.

#### 2.1.8. Coordination-Independent Multiple Schedule

##### Progressive-Delay Component

Two identical but independent progressive-delay schedules were in effect. Independently for each dyad member, after the elapse of their respective VI interval, the first keypeck initiated a delay period. At the end of the delay period, the reinforcer was presented independently for each dyad member without any additional response required for its presentation.

##### Yoked-Immediate-Reinforcement Component

Two independent sequences of IRIs generated from coordination-independent delayed reinforcement, one for each member of a dyad, were used to arrange two schedules of coordination-independent immediate reinforcement. For each member of the dyad, the first keypeck following the elapse of the corresponding yoked IRI was reinforced. Because two independent schedules were in effect during this component, one member of a dyad (Pigeon A) could complete its sequence of IRIs before its coactor (Pigeon B). In this case, the session terminated for Pigeon A, and the compartment was darkened by turning off the houselight and response keylight. The contingency, however, remained in effect in Pigeon B’s compartment until completion of its sequence of IRIs. After the session terminated for Pigeon A and its compartment was darkened, the session timer was paused while Pigeon B continued responding. Any responses emitted by Pigeon B thereafter were recorded but excluded from further analysis. Once Pigeon B completed its sequence of IRIs, the session terminated.

#### 2.1.9. Measurement

The coordination ratio was computed by dividing the number of coordinated responses by the total number of coordinated and uncoordinated responses in the coordination-dependent delay and immediate-reinforcement components. When computing coordination ratios for the coordination-independent delay and immediate-reinforcement components, however, keypecks emitted by one member of a dyad during reinforcer delivery to its coactor were excluded from the total number of responses. Also excluded were responses emitted by the remaining pigeon in the coordination-independent immediate-reinforcement component after the session had terminated for its coactor.

Additional analyses of coordination-dependent delayed and immediate reinforcement were conducted by examining coordinated responses per minute across successive interreinforcer intervals (IRIs), within-session changes in coordination postreinforcement pauses (PRPs) across successive IRIs, and obtained delays across successive IRIs.

Coordinated responses per minute across successive IRIs were calculated by dividing the number of coordinated responses within each IRI by the duration of that interval to assess changes in coordination rates across intervals.

Coordination PRPs across successive IRIs, defined as the time between reinforcer termination and the first coordinated response, were also computed across successive IRIs to assess how pauses following reinforcement changed across intervals.

The obtained delay across successive IRIs, defined as the time between the last coordinated response and reinforcer delivery, was also computed across successive IRIs to assess how experienced delays changed as programmed delays increased.

Break point was used as a measure of behavioral persistence under progressively increasing delays. For the coordination-dependent delayed-reinforcement component, a break point was defined as the last completed delay value before 5 min elapsed without a coordinated response. Because reinforcement in this component depended on coordinated responding, a single break point was obtained for each dyad. For the coordination-independent delayed-reinforcement component, sessions likewise terminated after 5 min without a coordinated response. However, because each member of the dyad was exposed to a separate and independent progressive-delay contingency, a break point was determined separately for each pigeon and was defined as the highest completed delay value attained by that pigeon prior to session termination.

Finally, reinforcers per minute were computed by dividing the total number of reinforcers by session duration in the coordination-dependent delay and immediate-reinforcement components. In the coordination-independent delay and immediate-reinforcement components, however, reinforcers per minute were computed separately for each member of a dyad.

### 2.2. Results

This section discusses the results of the two comparisons outlined in the Introduction. The first comparison examined the effects of delayed and immediate reinforcement on coordinated responding under coordination-dependent reinforcement. Primary analyses focused on overall coordination ratios and within-session changes in coordination rate across successive IRIs. The second comparison examined the persistence of coordinated responding under delayed reinforcement by comparing coordination-dependent and coordination-independent reinforcement conditions. All analyses were based on the final six sessions of each component. The number of sessions and obtained reinforcement rates for each component are summarized in Table 2. This table also served as a procedural check on the effectiveness of the yoked-control arrangements. In general, obtained reinforcement rates were comparable across delayed and yoked-immediate-reinforcement components, suggesting that differences in coordinated responding were not attributable to substantial differences in reinforcement frequency across components.

#### 2.2.1. Comparing Coordination-Dependent Delayed and Immediate Reinforcement

The analyses presented in this section compared coordinated responding maintained by delayed and immediate reinforcement within the Coordination-Dependent Multiple Schedule.

**Figure 3:**
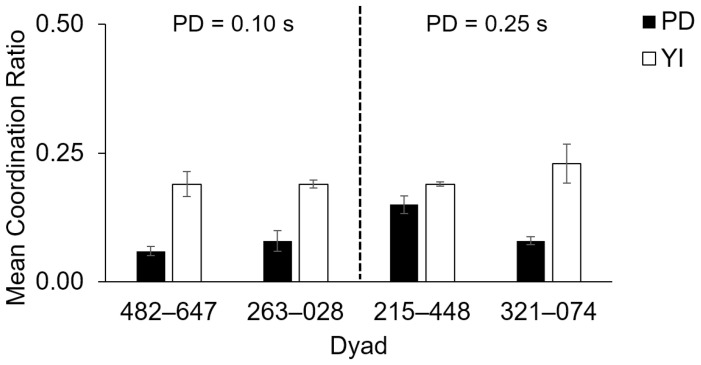
Mean coordination ratios for each dyad during coordination-dependent delayed (PD) and yoked-immediate (YI) reinforcement components in Experiment 1. Filled bars represent delayed reinforcement, and open bars represent yoked-immediate reinforcement. Dyads exposed to 0.10 s delay increments are shown to the left of the vertical dashed line, whereas dyads exposed to 0.25 s delay increments are shown to the right of the vertical dashed line. Error bars represent standard deviations computed from the last six sessions of each component.

Mean coordination ratios for each dyad are shown in Figure 3. Coordination ratios were consistently higher during immediate than delayed coordination-dependent reinforcement. The magnitude of this difference, however, varied across dyads (e.g., the difference was relatively modest for Dyad 215–448). This pattern is consistent with previous findings demonstrating that delays to reinforcement weaken operant behavior. A similar pattern was observed during coordination-independent reinforcement, with coordination ratios consistently higher during immediate than delayed reinforcement, although the magnitude of the difference also varied across dyads (see Supplementary Figure S1).

**Figure 4:**
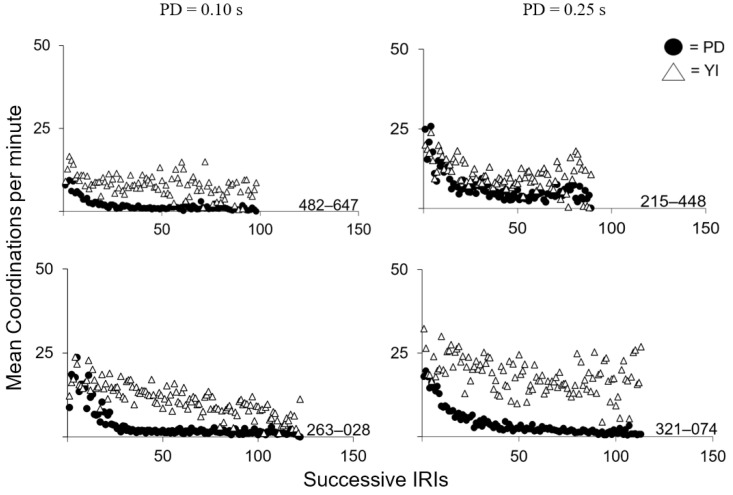
Coordination rates across successive interreinforcer intervals (IRIs) for each dyad during the coordination-dependent delayed (PD; filled circles) and yoked-immediate (YI; open triangles) reinforcement components in Experiment 1. Dyads exposed to 0.10 s delay increments are shown on the left, whereas dyads exposed to 0.25 s delay increments are shown on the right. Data points representing later IRIs were computed from fewer observations because fewer sessions reached those intervals.

Mean coordination rates across successive IRIs are shown in Figure 4. Coordination rates were high at the beginning of a session and declined as the session progressed during both coordination-dependent immediate- and delayed-reinforcement components. Coordination rates, however, generally were higher and more variable across intervals during immediate compared to delayed coordination-dependent reinforcement.

Additional analyses of relative changes in coordination across successive IRIs, coordination postreinforcement pauses (PRPs), and obtained delays are provided in Supplementary Figures S2–S4.

#### 2.2.2. Comparing Coordination-Dependent and Coordination-Independent Delayed Reinforcement

The analyses presented in this section compared the persistence of coordinated responding under coordination-dependent and coordination-independent delayed reinforcement.

**Figure 5:**
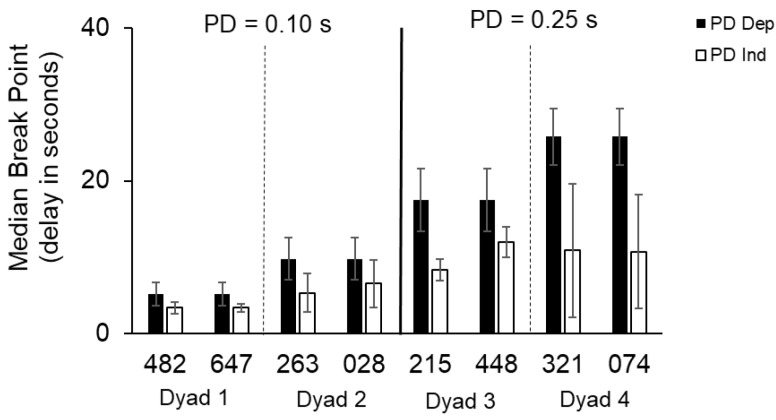
Median break points obtained during coordination-dependent (black bars) and coordination-independent (white bars) delayed reinforcement in Experiment 1. Vertical dashed lines separate pigeons within dyads, whereas the solid vertical line separates dyads exposed to 0.10 s and 0.25 s delay increments. Error bars represent interquartile ranges.

Median break points are shown in Figure 5. For the coordination-dependent delayed-reinforcement component, a break point was determined at the level of the dyad because reinforcement depended on coordinated responding. Consequently, the same break-point value is plotted for both members of a dyad in Figure 5. In contrast, break points in the coordination-independent delayed-reinforcement component were determined separately for each pigeon because each member of the dyad was exposed to an independent progressive-delay contingency and therefore could attain a different maximum completed delay value. Median, rather than mean, break points were examined to minimize the influence of outliers in the data. Break points were consistently higher for each pigeon during coordination-dependent compared to coordination-independent delayed reinforcement, indicating greater persistence of coordinated responding when reinforcement was contingent on coordination. Specifically, relative to coordination-independent delayed reinforcement, median break points during coordination-dependent delayed reinforcement were between 49% and 83% higher for pigeons exposed to 0.10 s delay increments and between 46% and 140% higher for pigeons exposed to 0.25 s delay increments (see Supplementary Figure S5).

### 2.3. Discussion

Experiment 1 examined the effects of progressively increasing delays of reinforcement on coordinated responding and the role of coordination–reinforcer dependency in the persistence of coordination under delayed reinforcement. Coordination ratios and coordination rates generally were higher during immediate- than delayed-reinforcement components when reinforcement depended on coordination, indicating that progressively increasing delays weakened coordinated responding. In addition, break points obtained during coordination-dependent delayed reinforcement were consistently higher than those obtained for either member of the dyad during coordination-independent delayed reinforcement, suggesting greater persistence of coordinated responding when reinforcement depended on coordination.

In the present experiment, no stimulus change accompanied delays in the coordination-dependent delayed-reinforcement component. The presence and absence of a stimulus change during the delay period, however, have been shown to differentially affect individual operant responding (e.g., Ferster, 1953; Richards, 1981; Schaal & Branch, 1988). Reilly and Lattal (2004), for instance, examined the effects of signaled and unsignaled progressively increasing delays of reinforcement on individual operant responding in pigeons and found that both response rates and break points were differentially affected by the presence of a delay-correlated stimulus. Specifically, higher response rates and higher break points were obtained during signaled relative to unsignaled delays of reinforcement, suggesting greater maintenance and persistence of responding when the delay period was accompanied by a stimulus change. It remains unclear, however, whether the presence or absence of a stimulus change during the delay period similarly affects coordination ratios, coordination rates, and the persistence of coordinated responding. Experiment 2 was designed to address this question.

## 3. Experiment 2

Experiment 2 examined whether the presence or absence of a delay-correlated stimulus differentially affected coordinated responding maintained under progressively increasing delays of coordination-dependent reinforcement.

### 3.1. Method

#### 3.1.1. Subjects

As described in Experiment 1.

#### 3.1.2. Apparatus

As described in Experiment 1.

#### 3.1.3. Experimental Design

Experiment 2 compared coordinated responding maintained under signaled and unsignaled progressively increasing delays of coordination-dependent reinforcement. Of primary interest was whether a stimulus change accompanying the delay period would differentially affect the maintenance and persistence of coordinated responding as delay duration increased. Two separate two-component multiple schedules of coordination-dependent reinforcement were arranged: an Unsignaled-Delay Multiple Schedule and a Signaled-Delay Multiple Schedule. Each multiple schedule consisted of a progressive-delay component followed by a yoked-immediate-reinforcement component.

#### 3.1.4. General Procedure

The general procedure was as described in Experiment 1, except that preliminary training consisted of 10 sessions of exposure to a VI 30 s schedule of coordination-dependent immediate reinforcement. Dyad pairings were the same as in Experiment 1. Each dyad was exposed to signaled and unsignaled progressively increasing delays of reinforcement in counterbalanced order, as shown in Table 3.

#### 3.1.5. Common Features of the Multiple Schedules

The alternation of components, minimum number of sessions associated with each component, stability criteria, and general arrangement of progressive-delay and yoked-immediate reinforcement were identical to those described for the Coordination-Dependent Multiple Schedule in Experiment 1.

As in Experiment 1, delay increments were adjusted across dyads to maintain responding across the progressive-delay arrangement. Delay increments for Dyads 215–448 and 321–074 were 0.30 s, whereas delay increments for the remaining dyads were 0.10 s.

##### Yoked-Immediate-Reinforcement Component

For both multiple schedules, the arrangement of the yoked-immediate-reinforcement component was similar to the coordination-dependent immediate-reinforcement component of Experiment 1, except that only the obtained VI-link intervals from the preceding progressive-delay component were yoked rather than the entire obtained interreinforcer interval (VI-link interval + programmed delay duration). Specifically, the obtained VI-link intervals from the progressive-delay component served as response-independent variable-time (VT) intervals in the yoked-immediate-reinforcement component. Following the elapse of the yoked VT interval, the programmed progressive-interval (PI) duration (either 10 s or 30 s) was initiated, following which the first coordinated response produced immediate reinforcement. Thus, a tandem yoked-VT PI schedule with PI durations of either 10 s or 30 s was arranged in the yoked-immediate-reinforcement component. This arrangement permitted direct comparison of coordinated responding during the initial-link schedule under delayed and immediate reinforcement while controlling for reinforcement rate and distribution across sessions and maintaining consistency with procedures commonly used in previous investigations of delayed reinforcement (e.g., Reilly & Lattal, 2004; Richards, 1981; Schaal & Branch, 1988).

#### 3.1.6. Unsignaled-Delay Multiple Schedule

##### Unsignaled-Delay Component

The arrangement of the unsignaled-delay component was identical to the coordination-dependent delayed-reinforcement component used in Experiment 1.

##### Yoked-Immediate-Reinforcement Component

After the elapse of the yoked interval, the first coordinated response resulted in immediate reinforcement delivery to both dyad members.

#### 3.1.7. Signaled-Delay Multiple Schedule

##### Signaled-Delay Component

The arrangement of the signaled-delay component was identical to the coordination-dependent delayed-reinforcement component used in Experiment 1, except that the entire delay period was accompanied by a darkening of the response keylights in each compartment while the houselight remained on. This darkening of the response keylights served as the delay-correlated stimulus signaling the delay period.

##### Yoked-Immediate-Reinforcement Component

After the elapse of the yoked interval, the first coordinated response resulted in immediate reinforcement delivery to both dyad members.

#### 3.1.8. Measurement

As described in Experiment 1.

### 3.2. Results

The analyses presented below were based on the final six sessions of each component. The number of sessions and reinforcers per minute in each component of the multiple schedules are shown in Table 4. In general, obtained reinforcement rates were comparable across delayed and yoked-immediate-reinforcement components within both multiple schedules. Reinforcement rates, however, generally were higher during the Signaled-Delay Multiple Schedule than during the Unsignaled-Delay Multiple Schedule.

**Figure 6:**
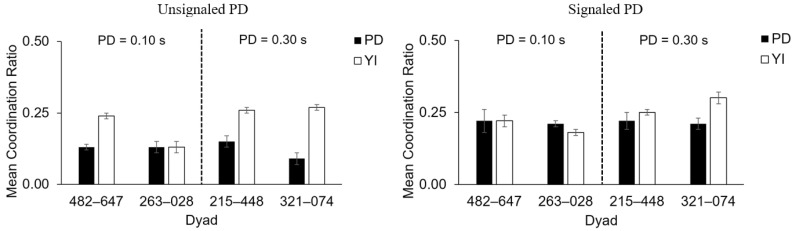
Mean coordination ratios during unsignaled and signaled delays of coordination-dependent reinforcement and their corresponding yoked-immediate (YI) reinforcement components for each dyad in Experiment 2. Filled bars represent delayed reinforcement, and open bars represent yoked-immediate reinforcement. Panels on the left depict unsignaled delays, whereas panels on the right depict signaled delays. Dyads exposed to 0.10 s delay increments are shown to the left of the vertical dashed line, whereas dyads exposed to 0.30 s delay increments are shown to the right of the vertical dashed line. Error bars represent standard deviations computed from the last six sessions of each component.

Mean coordination ratios during unsignaled and signaled delays of reinforcement and their corresponding yoked-immediate-reinforcement components are shown in Figure 6. For Dyads 482–647, 215–448, and 321–074, coordination ratios generally were higher during immediate reinforcement compared to unsignaled delayed reinforcement. In contrast, there was no substantial difference between unsignaled delayed and immediate reinforcement for Dyad 263–028.

Coordination ratios during signaled delayed reinforcement generally were comparable to those obtained during immediate reinforcement. For Dyad 482–647, for instance, coordination ratios were similar across components. For Dyad 263–028, coordination ratios were modestly higher during signaled delayed reinforcement than during immediate reinforcement. Conversely, for Dyads 215–448 and 321–074, coordination ratios were modestly higher during immediate reinforcement than during signaled delayed reinforcement.

**Figure 7:**
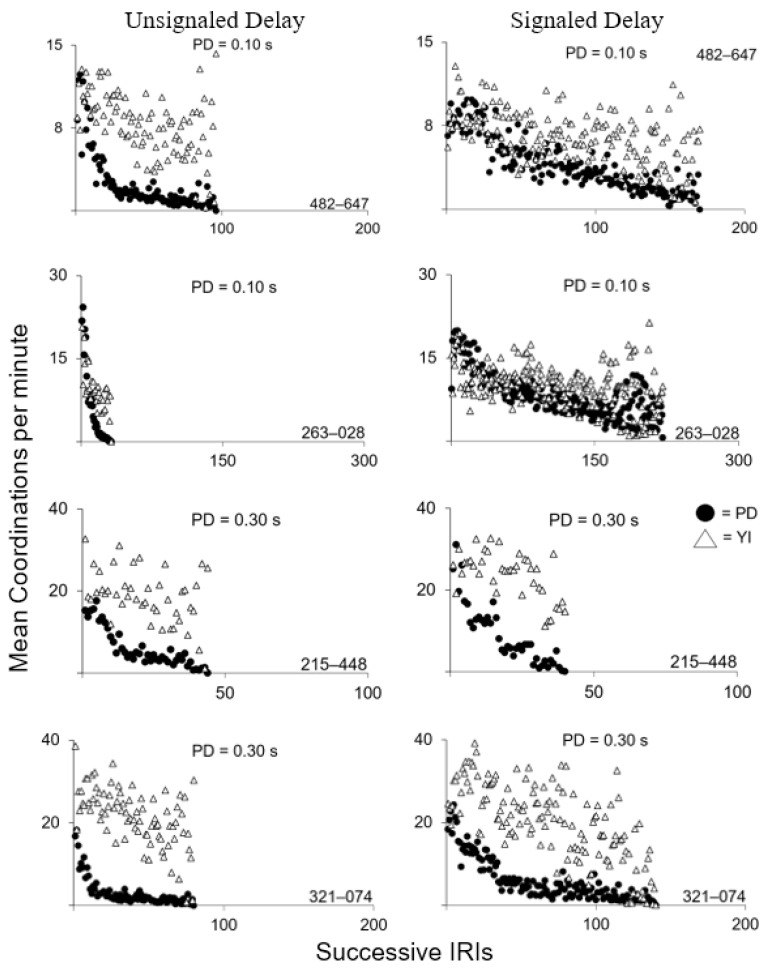
Coordination rates across successive interreinforcer intervals (IRIs) during unsignaled and signaled delays of coordination-dependent reinforcement and their corresponding yoked-immediate (YI) reinforcement components for each dyad in Experiment 2. Filled circles represent delayed reinforcement, and open triangles represent yoked-immediate reinforcement. Data points representing later IRIs were computed from fewer observations because fewer sessions reached those intervals.

Coordination rates across successive IRIs during unsignaled and signaled delay components and their corresponding yoked-immediate-reinforcement components are shown in Figure 7. Coordination rates were highest early in the session and declined as the session progressed under both delay and immediate-reinforcement components. Coordination rates, however, generally were lower during both delay components compared to their corresponding immediate-reinforcement components.

**Figure 8:**
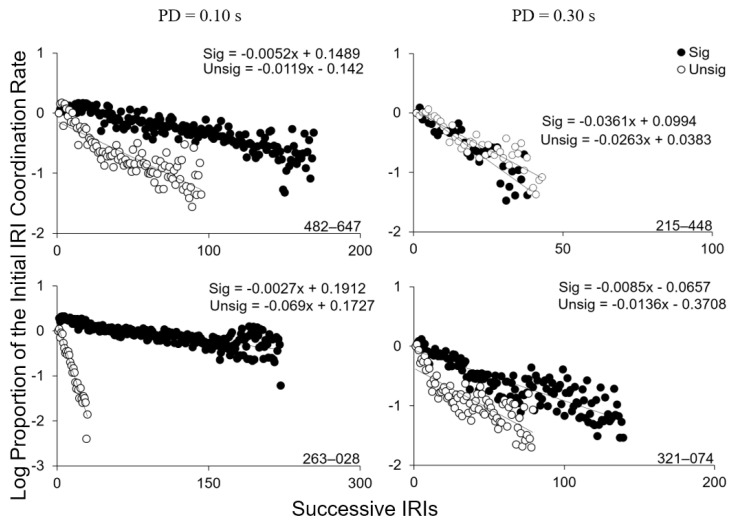
Log proportion of the initial interreinforcer interval (IRI) coordination rate across successive IRIs comparing signaled and unsignaled delays of coordination-dependent reinforcement for each dyad in Experiment 2. Log proportions were calculated by dividing the coordination rate in each successive IRI by the coordination rate obtained during the initial IRI and then transforming the resulting proportion using the logarithm function. Filled circles represent signaled delays and open circles represent unsignaled delays. Lines represent linear fits describing the rate of decline in coordination across the session.

To directly compare within-session changes in coordination across the two delay arrangements, coordination rates obtained during each successive IRI were expressed as a proportion of the coordination rate obtained during the initial IRI of the session, and the resulting proportions were log transformed. As shown in Figure 8, the decline in coordination across successive IRIs generally was steeper during the unsignaled compared to the signaled delay component for three of the four dyads (Dyads 482–647, 263–028, and 321–074).

**Figure 9:**
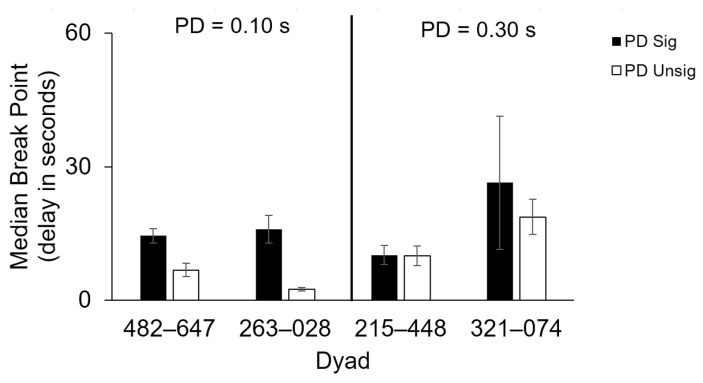
Median break points obtained during unsignaled (PD Unsig) and signaled (PD Sig) delays of coordination-dependent reinforcement in Experiment 2. Black bars represent signaled delays and white bars represent unsignaled delays. The solid vertical line separates dyads exposed to progressively increasing delay values of 0.10 s and 0.30 s. Error bars represent interquartile ranges.

Median break points obtained during unsignaled and signaled delays of coordination-dependent reinforcement are shown in Figure 9. Break points were higher during signaled compared to unsignaled delays for three of the four dyads, indicating greater persistence of coordinated responding when the delay period was accompanied by a stimulus change. Relative to unsignaled delays, median break points during signaled delays were 112%, 471%, and 40% higher for Dyads 482–647, 263–028, and 321–074, respectively (see Supplementary Figure S12). For Dyad 215–448, however, the median break point was 9% higher during unsignaled delays of reinforcement compared to signaled ones.

Additional analyses of coordination during initial- and terminal-link schedules, including coordination ratios and coordination rates during the VI and PT links, relative changes in coordination across successive IRIs, coordination rates during the initial-link schedule, coordination postreinforcement pauses (PRPs), and obtained delays are provided in Supplementary Figures S6–S11.

### 3.3. Discussion

Experiment 2 examined the effects of signaled and unsignaled delays of coordination-dependent reinforcement on coordinated responding. Coordination ratios and coordination rates generally were lower during unsignaled delayed compared to immediate reinforcement, replicating the response-reducing effects of delayed reinforcement observed in Experiment 1. In contrast, coordination maintained during signaled delays more closely resembled coordination maintained under immediate reinforcement, indicating that the presence of a stimulus change accompanying the delay period attenuated some of the disruptive effects of delayed reinforcement on coordinated responding. The differential effects of signaled and unsignaled delays were also reflected in measures of coordination persistence, with three of the four dyads exhibiting higher break points during signaled compared to unsignaled delayed reinforcement. Together, these findings demonstrate that coordinated responding, like individual operant behavior, is sensitive not only to delay duration but also to stimulus events correlated with delayed reinforcement. The broader implications of these findings for the analysis of coordinated behavior and delayed reinforcement are considered in the General Discussion.

## 4. General Discussion

To date, accumulated evidence indicates that coordination functions as an operant by showing that coordinated responding can be brought under the control of antecedent and consequent environmental events (e.g., de Carvalho et al., 2018; Lattal & Okouchi, 2023; Tan & Hackenberg, 2016; Todorov et al., 2020). Responses comprising an operant class are also characterized by their sensitivity to different parameters of reinforcement, including its rate, magnitude, and immediacy. Previous research, for instance, has shown that coordinated responding, like individual operant responding, is sensitive to changes in reinforcement rate (e.g., de Carvalho et al., 2020; dos Santos et al., 2023). The present investigation extends this literature by demonstrating that coordinated responding is similarly affected by delayed reinforcement and by variables related to delayed reinforcement, including progressively increasing delay duration and the presence or absence of a delay-correlated stimulus. These effects are discussed in the first three sections that follow, followed by consideration of the interpretation, limitations, and potential practical implications of the present findings.

### 4.1. Coordination-Reinforcer Dependency and Persistence of Coordination

The present investigation distinguished between the maintenance and persistence of coordinated responding during delayed reinforcement. Maintenance was assessed through measures such as coordination ratio and coordination rate during delayed reinforcement, whereas persistence was indexed by a break point under progressively increasing delays. Although both measures reflect the effects of delayed reinforcement on behavior, the break point provided an index of the extent to which coordinated responding continued under progressively worsening delay conditions. Thus, whereas coordination ratios and coordination rates reflected ongoing coordination during delayed reinforcement, the break point reflected resistance of coordinated responding to disruption produced by progressively increasing delays.

Experiment 1 examined whether coordination–reinforcer dependency differentially affected the persistence of coordinated responding during delayed reinforcement. Break points consistently were higher during coordination-dependent compared to coordination-independent delayed reinforcement, indicating greater persistence of coordinated responding when reinforcement depended on coordination. These findings extend previous demonstrations that coordinated responding is sensitive to the presence and absence of coordination–reinforcer dependency (e.g., de Carvalho et al., 2018; Lattal & Okouchi, 2023; Tan & Hackenberg, 2016; Todorov et al., 2020) by showing that coordination–reinforcer dependency also influences the persistence of coordinated responding under progressively increasing delays of reinforcement.

When comparing coordination-dependent and coordination-independent delayed reinforcement, reinforcement rates were not equated across conditions. The differential persistence of coordinated responding might be attributed, at least in part, to differences in reinforcement rate. For example, Nevin (1974) demonstrated that persistence of responding generally increases as a function of reinforcement rate. This interpretation, however, is inconsistent with the present findings because reinforcement rates were substantially higher during coordination-independent delayed reinforcement, whereas break points consistently were lower than those obtained during coordination-dependent delayed reinforcement (see Table 2). The greater persistence of coordinated responding observed during coordination-dependent delayed reinforcement more likely reflects the functional role of the coordination contingency itself rather than differences in reinforcement rate alone. Future investigations may clarify the relation between coordination–reinforcer dependency and behavioral persistence by systematically manipulating reinforcement rate, reinforcement magnitude, reinforcement probability, and disruptor variables commonly examined in analyses of behavioral momentum. Such analyses also may contribute to ongoing evaluations of behavioral momentum theory and the conditions under which its predictions are and are not supported (Craig, 2023).

### 4.2. Delay Gradients and Coordination

Delay gradients, defined as systematic changes in behavior as a function of increasing delays between responding and reinforcement, have been observed across a wide range of individual operant response topographies (e.g., Azzi et al., 1964; Galuska & Woods, 2005; Lattal & Metzger, 1994; Okouchi, 2009; Williams, 1976). A similar pattern was observed in Experiments 1 and 2, where operant responses defined across two organisms were higher when reinforcement was immediate and, like individual operant responses, decreased systematically with increasing delays of reinforcement. These findings are consistent with those of Reilly and Lattal (2004) with respect to within-session delay gradients of individual operants, as well as those of Sizemore and Lattal (1978), who reported that the rate of keypecking diminished substantially and proportionally as a function of increasing delay duration across sessions. Thus, delays engender coordination delay gradients that are comparable to delay gradients observed in individual operants (e.g., Lattal, 2010; Renner, 1964; Tarpy & Sawabini, 1974).

The changes in the coordination rates during the delay of reinforcement conditions might have been a result of progressively increasing the delay period in conjunction with changes in the rate and distribution of reinforcers occurring within the session. The yoking procedure, however, controlled for the changes in the rate and distribution of reinforcers between immediate and delay conditions. The difference in coordination rate observed between delay and immediate reinforcement conditions, therefore, can be attributed to the imposition of delays in the former condition. This conclusion was further supported by the within-session patterns of coordinated responding shown in Figure 4 and Figure 7, as well as the corresponding analyses of relative declines in coordination presented in Supplementary Figures S2 and S7.

The methodological arrangement of the delay and immediate reinforcement conditions in the present investigation can be contrasted with other investigations that have not equated the rate and distribution of reinforcers between immediate and delayed reinforcement conditions (e.g., Ferster, 1953; Morgan, 1972; Richards, 1981). Ferster (1953), for instance, used a VI 60 s schedule as an immediate reinforcement condition against which a delay condition consisting of a chained VI 60 s FT 60 s schedule was examined and found that keypecking was maintained at a higher rate with the former condition. In this instance, the lower rate of keypecking during the delay compared to the immediate reinforcement condition cannot be solely attributed to the imposition of delay alone, since the rate and distribution of reinforcers between these conditions were also unequal. Hence, the outcome of the present investigation provides additional support to the conclusion of Reilly and Lattal (2004) that a within-session progressively increasing delay of reinforcement is an effective procedure for examining the unconfounded effects of delays of reinforcement on operant responses. Future investigations may benefit from systematically manipulating delay magnitude and schedule parameters independently to further clarify the relation between delay gradients engendered by coordinated and individual operant behavior. For example, future studies could directly compare delay gradients obtained from coordinated and individual operant responses under otherwise identical reinforcement arrangements to determine whether coordinated behavior is differentially sensitive to delayed reinforcement.

### 4.3. Signaled Delay of Reinforcement and Coordination

The response-reducing effects of delayed reinforcement can be attenuated by imposing a stimulus change during the delay period (e.g., Ferster, 1953; Richards, 1981; Schaal & Branch, 1988). Richards (1981), for instance, compared the responding maintained during unsignaled and signaled delays of reinforcement and found that higher response rates were maintained during signaled compared to unsignaled delays. The primary dependent measure in Richards’ experiment was the maintenance of response rates under delayed reinforcement, whereas the present investigation additionally evaluated the persistence of coordinated responding using progressively increasing delays and break-point measures. Thus, the present findings extend previous analyses of signaled and unsignaled delayed reinforcement by demonstrating differential persistence of coordinated responding as delay duration progressively increased.

In Experiment 2, coordination persisted more during signaled than unsignaled delay of reinforcement for most dyads, a finding consistent with those reported by Reilly and Lattal (2004) for individual operants in pigeons. The differential effects of signaled and unsignaled delays, however, were not limited to the persistence of coordination. As in Experiment 1, the rate of coordination was high at the beginning of a session and substantially declined as the session progressed in both signaled and unsignaled delay conditions. The delay gradient slope, however, was steeper during unsignaled than signaled delays of reinforcement, showing that the presence or absence of a stimulus change also differentially affected the delay gradients engendered by delayed reinforcement (see Figure 8).

A caveat to the interpretation of the greater persistence of coordinated responding and the slower decline in coordination during signaled delay is that reinforcement rates generally were higher during the signaled-delay component relative to the unsignaled-delay component (see Table 4). Consequently, the greater persistence of coordinated responding and the less steep decline in coordination observed during signaled delay cannot be attributed exclusively to the presence of a delay-correlated stimulus. Reinforcement rate has been shown to influence the persistence of operant behavior (e.g., Nevin, 1974), and therefore differences in reinforcement frequency may have contributed to the observed differences between signaled and unsignaled delay conditions. Nevertheless, because the effects of signaled delay were observed across multiple measures of coordinated responding, the findings suggest that delay-correlated stimuli likely contributed to the observed differences.

Further support for this interpretation was provided by the coordination-ratio data. When comparing the coordination ratios during unsignaled delay and immediate reinforcement, coordination ratios were consistently higher during immediate reinforcement in both Experiments 1 and 2. Conversely, there was no systematic difference in coordination ratios between signaled delay and immediate reinforcement. The presentation of a stimulus change during the delay period, therefore, attenuated the effects of delay and, as shown in Experiment 2, produced behavior more comparable to that observed under immediate reinforcement (e.g., Lattal, 2010; Richards, 1981; Schaal & Branch, 1988).

Additional analyses of coordination PRPs, obtained delays, and responding across initial- and terminal-link schedules further suggested that signaled and unsignaled delays differentially affected the temporal organization of coordinated responding within sessions. Coordination PRPs generally were higher during unsignaled delay relative to immediate reinforcement in both Experiments 1 and 2 (see Supplementary Figures S3 and S10), whereas PRPs during signaled delay were not systematically different from those observed during immediate reinforcement (see Supplementary Figure S10). Obtained delays generally tracked programmed delays in both conditions, although variability increased more systematically during unsignaled relative to signaled delay (see Supplementary Figures S4 and S11). Coordination ratios generally were not systematically different between the initial- and terminal-link schedules during unsignaled delay, whereas coordination ratios generally were higher during the initial link relative to the terminal link during signaled delay (see Supplementary Figure S6). Coordination rates, however, generally were higher during the terminal link relative to the initial link during unsignaled delay, whereas coordination rates were higher during the initial link relative to the terminal link during signaled delay (see Supplementary Figure S9). Although these measures were not the primary focus of the present investigation, the findings are generally consistent with previous analyses of individual operant behavior maintained under delayed and signaled reinforcement procedures (e.g., Ferster, 1953; Reilly & Lattal, 2004; Richards, 1981; Schaal & Branch, 1988) and further support the interpretation of coordinated responding as sensitive to variables known to influence individual operants.

Future studies could equate reinforcement rates across signaled and unsignaled delay conditions or manipulate signal presence and reinforcement rate independently to isolate their respective contributions to coordinated responding. In addition, future research may further clarify the functions of delay-correlated stimuli by systematically manipulating signal properties, signal duration, and stimulus–reinforcer relations during delayed reinforcement of coordinated responding. For example, investigators could compare signals that differ in duration or salience, or arrange signals that either consistently or inconsistently predict reinforcement delivery, to determine which stimulus characteristics most effectively attenuate the disruptive effects of delayed reinforcement on coordinated responding.

### 4.4. Signaled Delay of Reinforcement and Delay Reduction Theory

The differential effects of signaled and unsignaled delays observed in the present investigation are generally consistent with Delay Reduction Theory (Fantino, 1969; Fantino & Dunn, 1993). According to this account, stimuli correlated with a reduction in time to reinforcement may acquire conditioned reinforcing properties. Thus, during the signaled delay conditions, the onset of the delay-correlated stimulus may have functioned as a conditioned reinforcer that bridged the temporal gap between coordinated responding and primary reinforcement delivery. By contrast, no such stimulus accompanied the delay period in the unsignaled delay conditions.

Interpretations of this sort have commonly been advanced in analyses of concurrent-chains procedures, where stimuli correlated with terminal-link schedules acquire conditioned reinforcing properties as a function of the reduction in time to reinforcement they signal (e.g., Fantino, 1969; Squires & Fantino, 1971). In the present investigation, the onset of the delay-correlated stimulus may have similarly functioned as a discriminative stimulus, signaling an increased probability or temporal proximity of reinforcement relative to the initial-link schedule. In this way, the delay signal may have attenuated the disruptive effects of delayed reinforcement by effectively reducing the functional delay to reinforcement.

Additional analyses of responding occurring during the delay period itself may help clarify whether delay-correlated stimuli function primarily as conditioned reinforcers, discriminative stimuli, or both during coordinated responding. In the present investigation, coordination rates during the delay period generally were low (see Supplementary Figure S9). By contrast, studies using delay-correlated stimuli have sometimes reported substantial responding during the delay period (e.g., Schaal & Branch, 1988). These differences suggest that delay-correlated stimuli may serve multiple behavioral functions and that the specific properties of such stimuli may influence those functions. Future studies could directly evaluate responding during the delay interval and manipulate the relation between delay-correlated stimuli and reinforcement delivery to determine the behavioral functions served by these stimuli. Such analyses may contribute to a more complete account of how delay-correlated stimuli attenuate the disruptive effects of delayed reinforcement on coordinated responding.

### 4.5. Limitations of the Present Analysis and Potential Future Directions

In the prior investigations, reinforcement rates between coordination-dependent and coordination-independent reinforcement have been equated to assess the effects of the presence and absence of coordination-reinforcer dependency on coordination (e.g., de Carvalho et al., 2018; Lattal & Okouchi, 2023; Tan & Hackenberg, 2016; Todorov et al., 2020). In the present investigation, however, the reinforcement rates between these aforementioned conditions were not equated. This was done to address how the persistence of coordination, rather than the ratio or rate of coordination, was impacted during coordination-dependent and coordination-independent delayed reinforcement. Because coordination ratio and rate are the most widely used measures to evaluate the effects of the coordination-reinforcer dependency, it is also important to determine how these measurements are impacted by coordination-dependent and coordination-independent delayed reinforcement. One way to address this issue is to arrange coordination-independent delayed reinforcement that is yoked to coordination-dependent delayed reinforcement. With this arrangement, reinforcement rate as a confounding variable can be eliminated, and the impact of coordination-dependent and coordination-independent delayed reinforcement on coordination ratio and rate can be directly examined. This arrangement of delayed coordination-dependent and coordination-independent reinforcement is yet to be examined.

Another limitation concerns the use of a VI 20 s schedule as the initial link that was followed by a PT-x (either 0.10 s, 0.25 s, or 0.30 s) schedule as the terminal link when arranging progressively increasing delays of coordination-dependent and coordination-independent reinforcement. One consequence of using the VI 20 s schedule as the initial link was that the increase in the interreinforcer intervals (VI and PT link) occurred over the long run and not necessarily from one reinforcer presentation to the next. Hence, the progressively increasing delay of coordination-dependent and coordination-independent reinforcement arranged in the present investigation can be described as quasiprogressive (for a more detailed discussion on this topic, see Reilly & Lattal, 2004). To address this issue, it is possible to use an FI rather than a VI schedule as the initial link that is followed by a PT schedule. The use of the FI schedule as the initial link would ensure that the interreinforcer interval systematically increases from one reinforcer delivery to the next. It is important to note that Reilly and Lattal (2004) compared progressive-delay schedules that had either a VI or an FI schedule as the initial link, but they did not observe any substantial difference in keypecking engendered by these arrangements. It, however, remains to be investigated how the use of VI and FI schedules as the initial link affects coordination.

Another limitation concerns the delay-correlated stimulus used during the signaled-delay component in Experiment 2. When examining the effects of signaled delay of reinforcement, the change in keylight color has often been used to accompany the delay period (e.g., Ferster, 1953; Morgan, 1972; Pierce et al., 1972). The use of a keylight color change to signal the delay period, in turn, has been shown to result in substantial responding occurring throughout the delay period (e.g., Schaal & Branch, 1988). In Experiment 2 of the present investigation, however, the darkening of the response keylights in each compartment was used as a signal in the signaled delay of reinforcement. The use of a darkened keylight as a signal during the delay period, however, can result in a substantial reduction in responding during the delay period (e.g., Harris et al., 2012). Thus, the overall reduction in coordination observed during the delay period in the signaled delay of reinforcement might be specifically attributed to the use of darkening of the response keylights as a signal rather than signaling the delay period in general (see Supplementary Figure S9).

The use of darkening of the response keylights as a signal also could have impacted the outcome of Experiment 2 for Dyad 215–448. After session termination during signaled delay condition, it was common to observe Pigeon 215 facing toward the opposite direction of the response panel whenever the investigator removed the pigeon from the operant chamber. If the darkening of the response keylights functioned as an aversive stimulus that led to the cessation of responding in Pigeon 215, it could potentially explain why no systematic difference between unsignaled and signaled delay was observed in this dyad. Although this interpretation was initially based on informal observation, additional data were collected in a follow-up condition for this dyad. When a blackout (i.e., turning off both the houselight and keylights) was used as the delay signal, persistence of coordination was higher in the signaled than in the unsignaled delay condition (see Supplementary Figure S13). Analysis of within-session changes further showed that coordination declined across successive IRIs in both conditions, but the rate of decline was steeper in the unsignaled condition than in the signaled (blackout) condition (see Supplementary Figure S14). These findings suggest that the specific properties of delay-correlated stimuli may influence coordinated responding. They also raise the possibility that individual learning histories may interact with the effects of delay-correlated stimuli, resulting in different patterns of responding across subjects. The interpretation that darkening of the response keylights functioned as an aversive stimulus for Pigeon 215 differs from the delay-reduction and conditioned-reinforcement account discussed earlier. The two explanations, however, are not necessarily mutually exclusive. Delay-correlated stimuli may serve multiple behavioral functions depending on their specific properties and an organism’s prior learning history. For example, a delay-correlated stimulus may function as a conditioned reinforcer for some subjects while simultaneously exerting aversive or response-suppressive effects for others. Further systematic investigation is needed to clarify these possibilities.

Another limitation was associated with challenges in identifying appropriate parameters for increasing delay values while maintaining coordination. In Reilly and Lattal (2004), for instance, the progressively increasing delay value was set to 2 s to maintain keypecking. In the present investigation, however, progressively increasing delay values were set to less than a second (i.e., 0.10 s, 0.25 s, or 0.30 s) to maintain coordination. Moreover, the delay increments had to be adjusted across dyads to sustain coordinated responding. Identifying appropriate parameters for progressively increasing delay values, in turn, took considerable time (approximately 3 months). The relatively small magnitude of the delay increments and the need to individualize them across dyads may suggest that maintaining coordination is more sensitive to changes in delay duration than are individual operants.

A final limitation involved the use of the same discriminative stimuli across multiple schedule arrangements. Although the stimuli consistently signaled the relation between coordinated responding and reinforcement within each schedule arrangement, reuse of the same stimuli across conditions may have weakened discriminative control or permitted carryover effects across successive schedule arrangements. Future investigations should examine whether the use of distinct discriminative stimuli across experimental conditions alters the acquisition and maintenance of coordinated responding under delayed reinforcement. Collectively, these directions may help clarify the extent to which coordinated responding is governed by the same behavioral processes known to influence individual operant behavior and may contribute to a more comprehensive analysis of social coordination as operant behavior.

### 4.6. Implications for Social Coordination in Applied Contexts

The present findings also may have implications for understanding the acquisition and maintenance of socially coordinated behavior in applied settings. Coordinated responding is often an important component of educational, therapeutic, and vocational interventions involving human participants, including interventions designed to establish cooperative play, peer interaction, joint activity engagement, and other socially coordinated repertoires (e.g., McKeown et al., 2021; Ziegler & Morrier, 2022). Although these interventions typically rely on socially mediated reinforcement, the present findings suggest that delays between coordinated responding and reinforcement delivery may disrupt the maintenance of coordinated behavior, whereas stimuli correlated with delayed reinforcement may mitigate these disruptive effects. From an applied perspective, arranging signals correlated with delayed reinforcement may support the persistence of coordinated responding when immediate reinforcement is impractical or unavailable. Such signals may help bridge delays to reinforcement delivery and thereby contribute to the maintenance of socially coordinated behavior over time.

### 4.7. Conclusions

Skinner (1953) suggested that social behavior is an operant. One characteristic of operant behavior is its sensitivity to different parameters of reinforcement, such as frequency, magnitude, and delay. Delay of reinforcement, for instance, has been shown to reduce operant response rates (Lattal, 2010; Renner, 1964; Tarpy & Sawabini, 1974). The present investigation contributes to our understanding of social operants by demonstrating that disruption in coordination-reinforcer temporal contiguity also has coordination rate-reducing effects. The coordination-rate-reducing effects of delays, in turn, can be mitigated by imposing a stimulus change during the delay period, hence providing support to the assertion that social operants are impacted by delay of reinforcement like any other operant response confined to the behavior of a single individual. 

## Figures and Tables

**Table 1:** Order of exposure, multiple-schedule arrangements, schedule components, and keylight colors for each dyad in Experiment 1.

Dyad	Order of Exposure	Multiple Schedule	Component	Key Color (A/B)
482–647	First	Coordination Independent	Progressive Delay	Blue/Orange
			Yoked Immediate	Yellow/Red
	Second	Coordination Dependent	Progressive Delay	Yellow/Red
			Yoked Immediate	Blue/Orange
263–028	First	Coordination Dependent	Progressive Delay	Yellow/Red
			Yoked Immediate	Blue/Orange
	Second	Coordination Independent	Progressive Delay	Blue/Orange
			Yoked Immediate	Yellow/Red
215–448	First	Coordination Dependent	Progressive Delay	Yellow/Red
			Yoked Immediate	Blue/Orange
	Second	Coordination Independent	Progressive Delay	Blue/Orange
			Yoked Immediate	Yellow/Red
321–074	First	Coordination Independent	Progressive Delay	Blue/Orange
			Yoked Immediate	Yellow/Red
	Second	Coordination Dependent	Progressive Delay	Yellow/Red
			Yoked Immediate	Blue/Orange

Note. Keylight colors indicate the stimulus assignments in Compartments A and B, respectively.

**Table 2:** Number of sessions and mean reinforcers per minute per component for each dyad during the coordination-dependent and coordination-independent multiple schedules of Experiment 1.

		Coordination-Dependent Multiple Schedule				Coordination-Independent Multiple Schedule			
Dyad	Pigeon	Component 1 (PD)		Component 2 (YI)		Component 1 (PD)		Component 2 (YI)	
		Reinforcers/min	Sessions	Reinforcers/min	Sessions	Reinforcers/min	Sessions	Reinforcers/min	Sessions
1	482	0.77	20	0.70	20	2.05	23	2.00	23
	647	0.77	20	0.70	20	1.96	23	1.88	23
2	263	0.75	32	0.82	32	1.72	20	1.64	20
	028	0.75	32	0.82	32	2.03	20	1.88	20
3	215	0.99	23	0.89	23	1.36	24	1.32	24
	448	0.99	23	0.89	23	1.99	24	1.95	24
4	321	0.86	20	0.82	20	1.69	22	1.66	22
	074	0.86	20	0.82	20	1.69	22	1.65	22

Note. Mean reinforcers per minute are based on the last six sessions of each component.

**Table 3:** Order of exposure, multiple-schedule arrangements, schedule components, and keylight colors for each dyad in Experiment 2.

Dyad	Order of Exposure	Multiple Schedule	Component	Key Color (A/B)
482–647	First	Unsignaled Delay	Progressive Delay	Blue/Orange
			Yoked Immediate	Yellow/Red
	Second	Signaled Delay	Progressive Delay	Yellow/Red
			Yoked Immediate	Blue/Orange
263–028	First	Signaled Delay	Progressive Delay	Yellow/Red
			Yoked Immediate	Blue/Orange
	Second	Unsignaled Delay	Progressive Delay	Blue/Orange
			Yoked Immediate	Yellow/Red
215–448	First	Signaled Delay	Progressive Delay	Yellow/Red
			Yoked Immediate	Blue/Orange
	Second	Unsignaled Delay	Progressive Delay	Blue/Orange
			Yoked Immediate	Yellow/Red
321–074	First	Unsignaled Delay	Progressive Delay	Blue/Orange
			Yoked Immediate	Yellow/Red
	Second	Signaled Delay	Progressive Delay	Yellow/Red
			Yoked Immediate	Blue/Orange

Note. Keylight colors indicate the stimulus assignments in Compartments A and B, respectively.

**Table 4:** Number of sessions and mean reinforcers per minute per component for each dyad during each multiple schedule of Experiment 2.

	Unsignaled-Delay Multiple Schedule				Signaled-Delay Multiple Schedule			
Dyads	Component 1 (PD)		Component 2 (YI)		Component 1 (PD)		Component 2 (YI)	
	Reinforcers/min	Sessions	Reinforcers/min	Sessions	Reinforcers/min	Sessions	Reinforcers/min	Sessions
482–647	0.86	20	0.80	20	1.38	20	1.21	20
263–028	0.96	23	0.84	23	1.69	20	1.40	20
215–448	1.13	20	1.09	20	1.34	20	1.30	20
321–074	0.88	20	0.86	20	1.29	20	1.22	20

## Data Availability

The original data presented in the study are openly available in the Open Science Framework at https://doi.org/10.17605/OSF.IO/MA59H.

## Supplementary Materials

The following supporting information can be downloaded at: https://www.mdpi.com/article/10.3390/bs16060967/s1, Figure S1: Mean coordination ratios for each dyad during the progressive-delay and yoked-interval components of coordination-independent reinforcement in Experiment 1; Figure S2: Log proportion of the initial interreinforcer interval (IRI) coordination rate across successive IRIs under coordination-dependent delayed and yoked-immediate reinforcement in Experiment 1; Figure S3: Mean coordination postreinforcement pauses (PRPs) across successive IRIs in Experiment 1; Figure S4: Mean obtained delays across successive IRIs under coordination-dependent delayed reinforcement in Experiment 1; Figure S5: Percent change in median break points during coordination-dependent relative to coordination-independent delayed reinforcement in Experiment 1; Figure S6: Mean coordination ratios during the VI and PT links of unsignaled and signaled delays of coordination-dependent reinforcement in Experiment 2; Figure S7: Log proportion of the initial IRI coordination rate across successive IRIs under unsignaled and signaled delays of coordination-dependent reinforcement and their corresponding yoked-immediate reinforcement in Experiment 2; Figure S8: Coordination rates during the initial-link schedule under unsignaled and signaled delays of coordination-dependent reinforcement and their corresponding yoked-immediate reinforcement in Experiment 2; Figure S9: Mean coordination rates during the VI and PT links of unsignaled and signaled delays of coordination-dependent reinforcement in Experiment 2; Figure S10: Mean coordination postreinforcement pauses across successive IRIs under unsignaled and signaled delays of coordination-dependent reinforcement and their corresponding yoked-immediate reinforcement in Experiment 2; Figure S11: Mean obtained delays across successive IRIs under unsignaled and signaled delays of coordination-dependent reinforcement in Experiment 2; Figure S12: Percent change in median break points during signaled relative to unsignaled delays of coordination-dependent reinforcement in Experiment 2; Figure S13: Median break points during signaled and unsignaled delays of coordination-dependent reinforcement for Dyad 215–448 in a follow-up condition; Figure S14: Log proportion of the initial IRI coordination rate across successive IRIs for Dyad 215–448 during signaled and unsignaled delays of coordination-dependent reinforcement in a follow-up condition.
