# Supplementary material for: Social Coordination Maintained by Progressive Delay of Coordination-Dependent Reinforcement
Source: Behav Sci (Basel). 2026 Jun 11;16(6):967. doi: 10.3390/bs16060967 (PMC13295747; doi:10.3390/bs16060967)
Supplement: Supplementary file 1 [file behavsci-16-00967-s001.zip › behavsci-4243772-supplementary.pdf]

Supplementary Materials: Social Coordination Maintained by Progressive Delay of Coordination-Dependent Reinforcement

Supplementary Figures

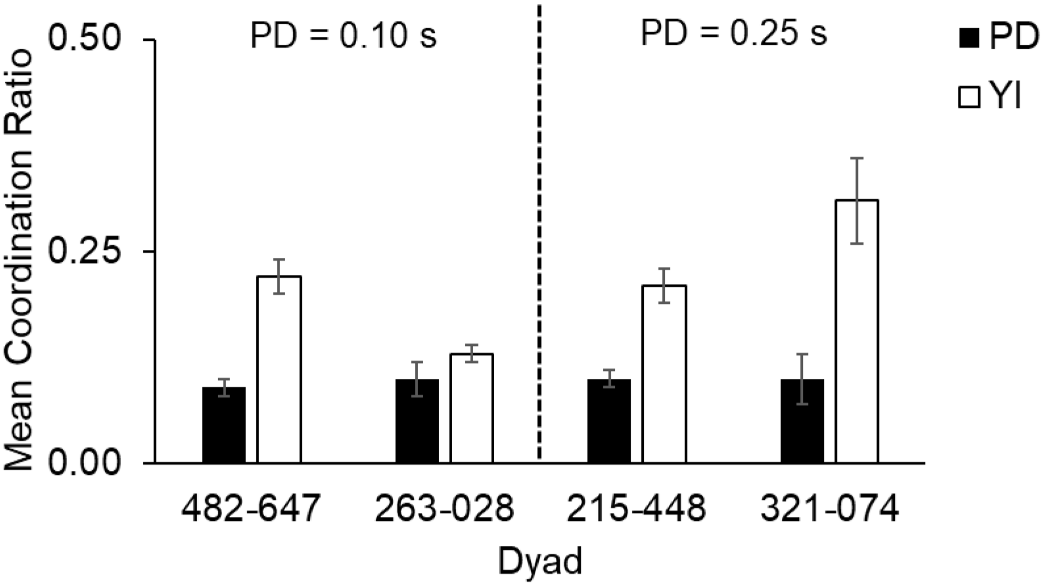

Figure S1: Mean coordination ratios for each dyad during the progressive-delay (black bars) and yoked-interval (white bars) components of coordination-independent reinforcement in Experiment 1. Coordination ratios were computed from the last six sessions of each component. The vertical dashed line separates dyads exposed to 0.10-s delay increments from those exposed to 0.25-s delay increments. Error bars represent standard deviations computed from the last six sessions of each component.

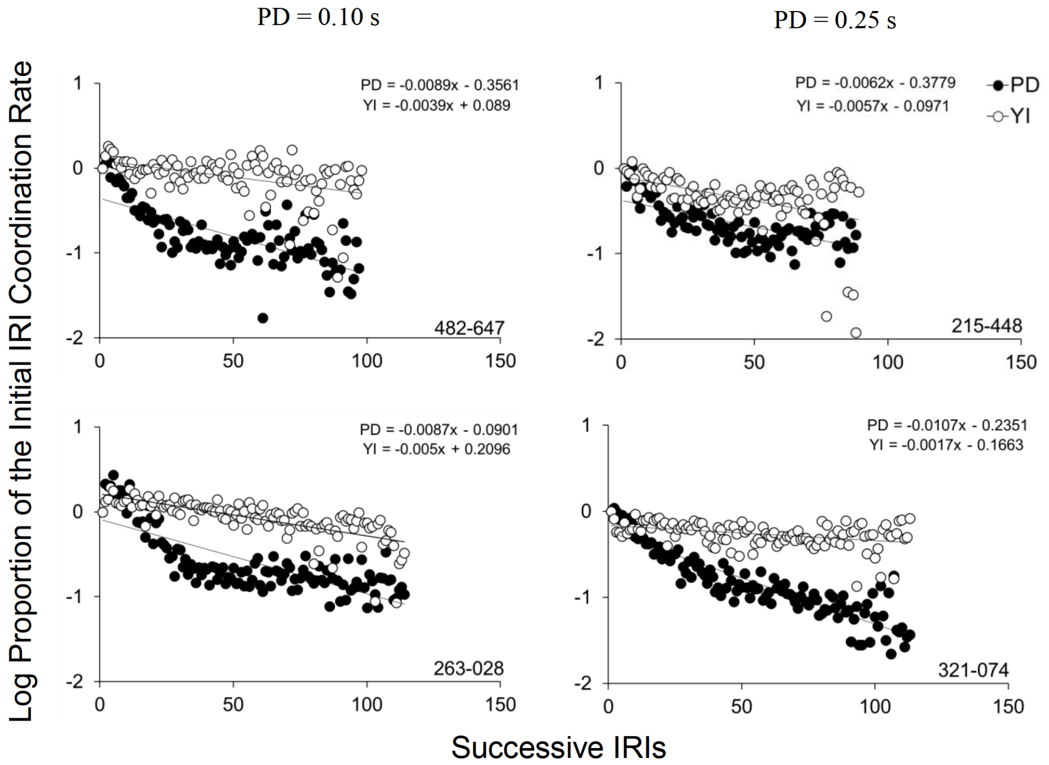

Figure S2: Log proportion of the initial interreinforcer interval (IRI) coordination rate across successive IRIs for each dyad under coordination-dependent delayed reinforcement (closed circles) and yoked-immediate (YI; open circles) reinforcement in Experiment 1. Log proportions were calculated by dividing the coordination rate in each successive IRI by the coordination rate in the initial IRI and then transforming the resulting proportion using the logarithm function. Dyads exposed to 0.10-s delay increments are shown on the left, whereas dyads exposed to 0.25-s delay increments are shown on the right. Data points representing later IRIs were computed from fewer observations because fewer sessions reached those intervals.

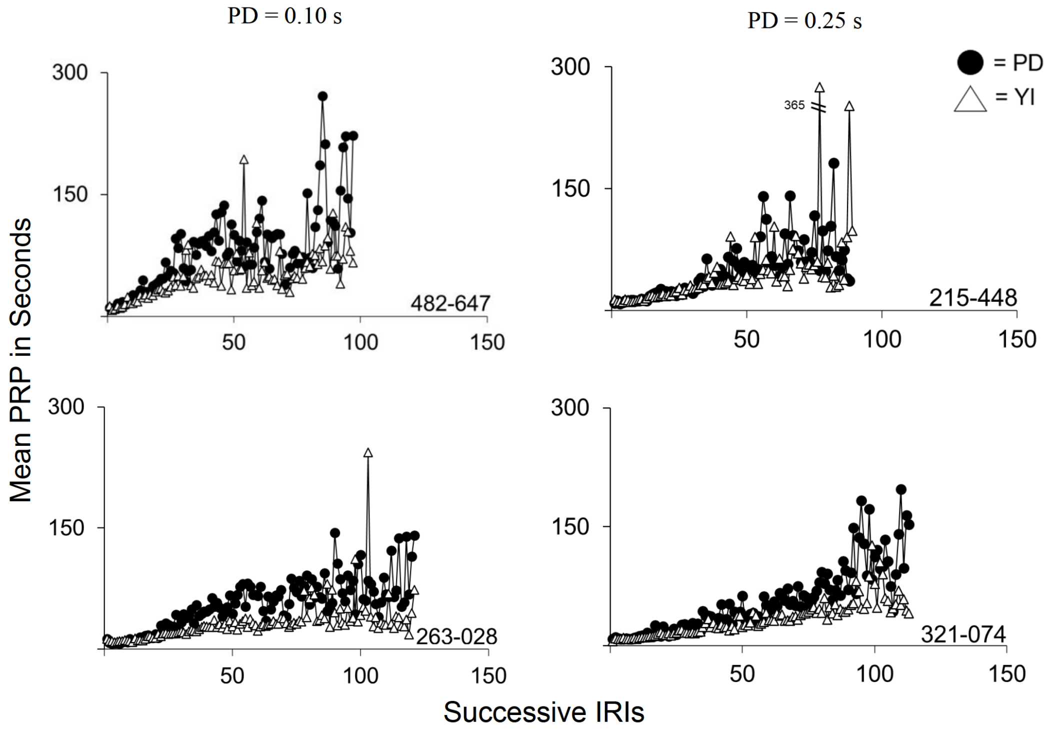

Figure S3: Mean coordination postreinforcement pauses (PRPs) across successive interreinforcer intervals (IRIs) for each dyad under coordination-dependent delayed reinforcement (closed circles) and yoked-immediate (YI; open triangles) reinforcement in Experiment 1. Dyads exposed to 0.10-s delay increments are shown on the left, whereas dyads exposed to 0.25-s delay increments are shown on the right. Data points representing later IRIs were computed from fewer observations because fewer sessions reached those intervals.

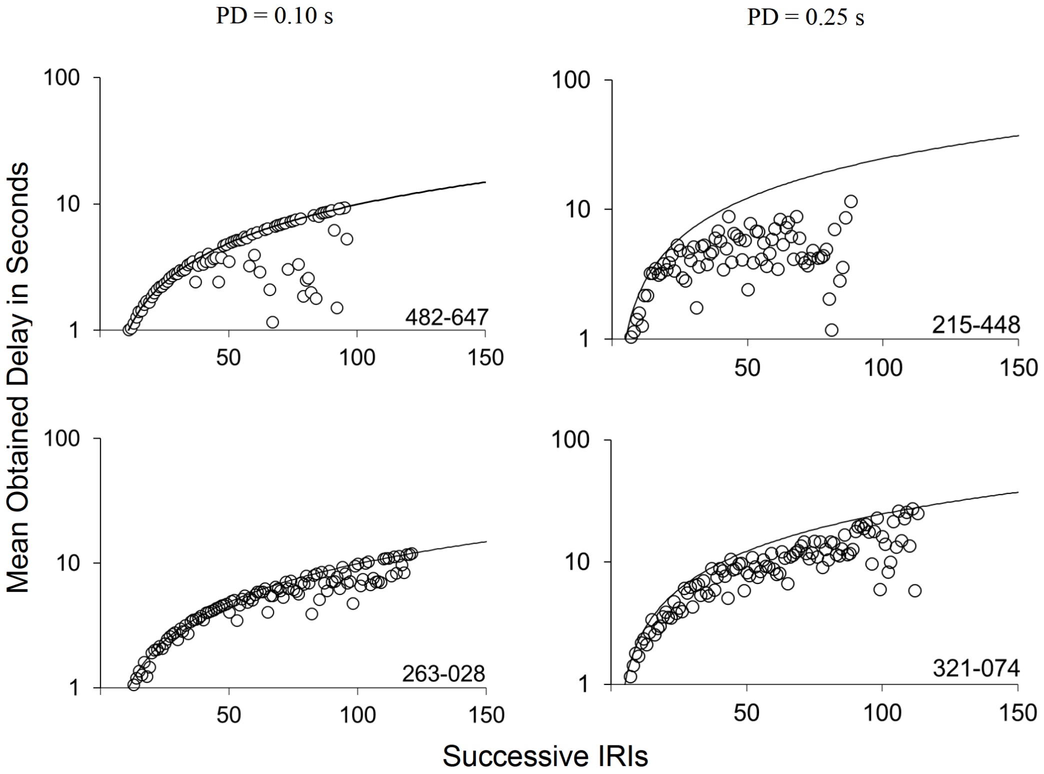

Figure S4: Mean obtained delays across successive interreinforcer intervals (IRIs) for each dyad under coordination-dependent delayed reinforcement in Experiment 1. Obtained delays are shown as open circles and programmed delays are shown as solid lines. The y-axis is plotted on a logarithmic scale. Dyads exposed to 0.10-s delay increments are shown on the left, whereas dyads exposed to 0.25-s delay increments are shown on the right. Data points representing later IRIs were computed from fewer observations because fewer sessions reached those intervals.

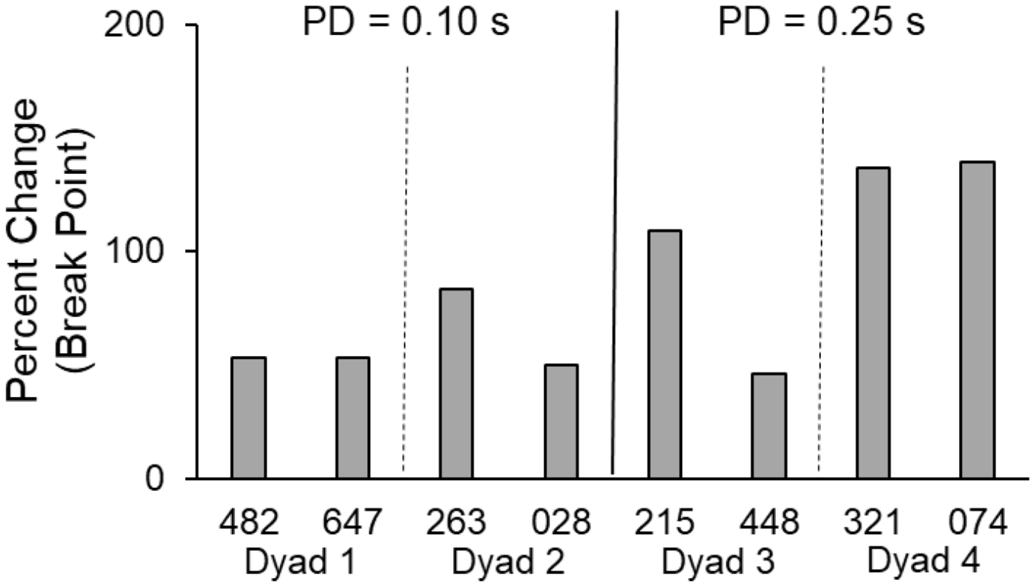

Figure S5: Percent change in median break points during coordination-dependent relative to coordination-independent delayed reinforcement for each pigeon in Experiment 1. Percent change was calculated by subtracting the median break point obtained during coordination-independent delayed reinforcement from the median break point obtained during coordination-dependent delayed reinforcement, dividing the difference by the coordination-independent value, and multiplying by 100. Percent change values were computed from the last six sessions of each component. Vertical dashed lines separate pigeons within dyads, and the solid vertical line separates dyads exposed to 0.10-s and 0.25-s delay increments.

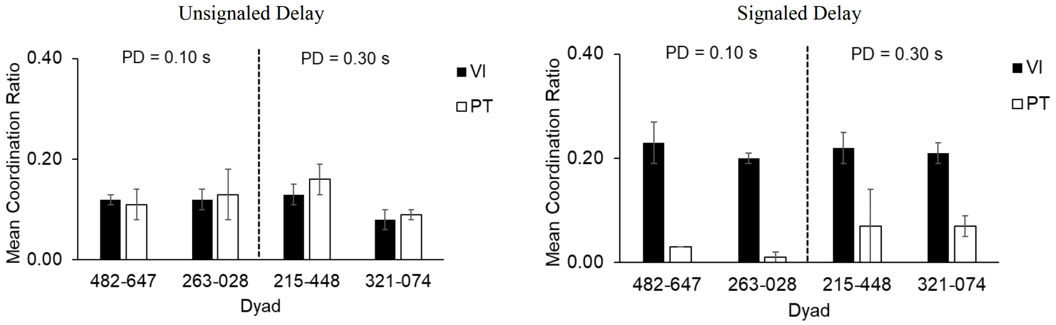

Figure S6: Mean coordination ratios during the VI and PT links of unsignaled and signaled delays of coordination-dependent reinforcement in Experiment 2. Filled bars represent coordination ratios during the VI link and open bars represent coordination ratios during the PT link. Panels on the left depict unsignaled delays, whereas panels on the right depict signaled delays. Dyads exposed to 0.10-s delay increments are shown to the left of the vertical dashed line, whereas dyads exposed to 0.30-s delay increments are shown to the right of the vertical dashed line. Data represent means from the last six sessions of each component.

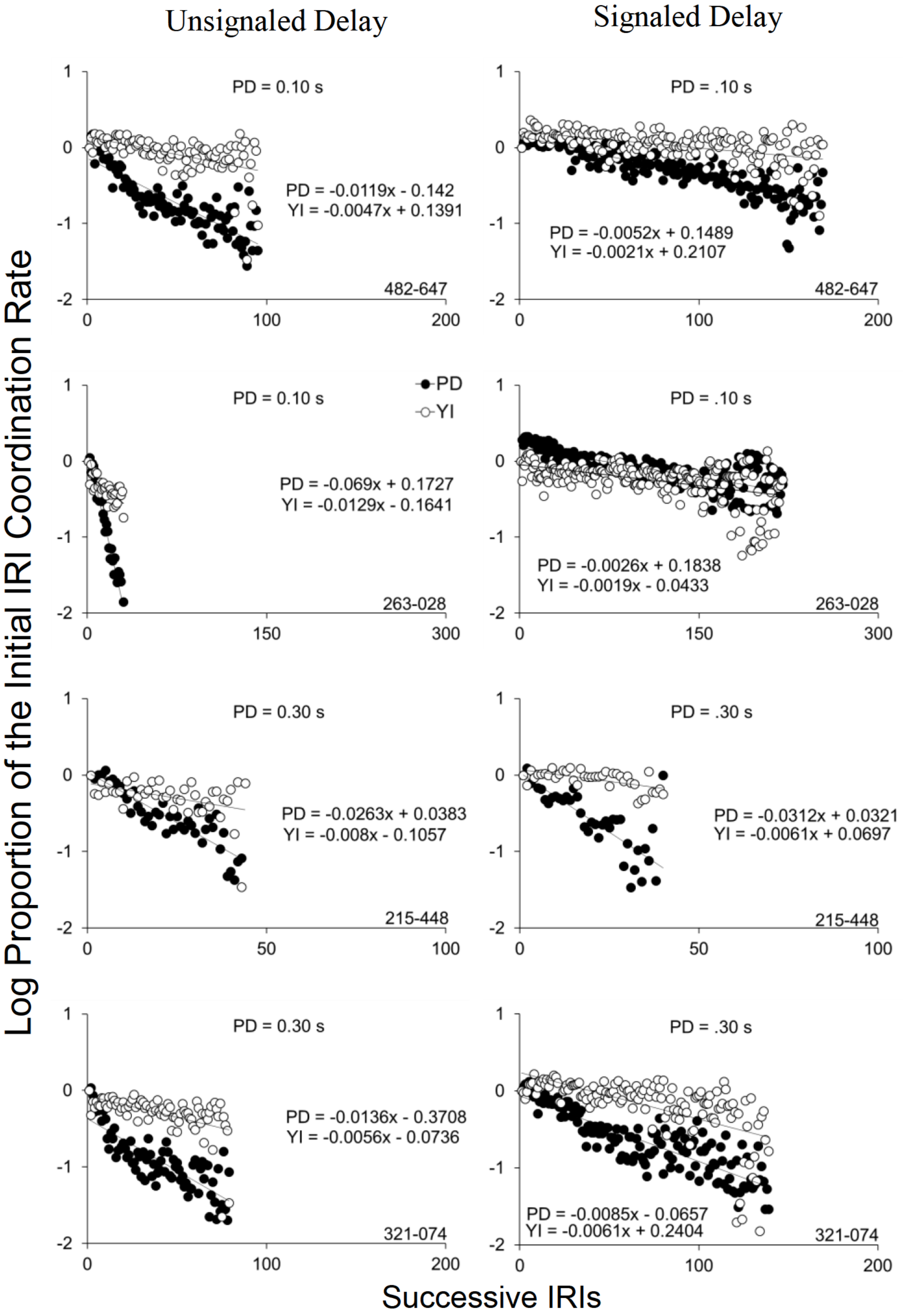

Figure S7: Log proportion of the initial interreinforcer interval (IRI) coordination rate across successive IRIs under unsignaled and signaled delays of coordination-dependent reinforcement and their corresponding yoked-immediate reinforcement in Experiment 2. Log proportions were calculated by dividing the coordination rate in each successive IRI by the coordination rate in the initial IRI and then transforming the resulting proportion using the logarithm function. Closed circles represent delayed-reinforcement conditions and open circles represent yoked-immediate-reinforcement conditions. Data points representing later IRIs were computed from fewer observations because fewer sessions reached those intervals.

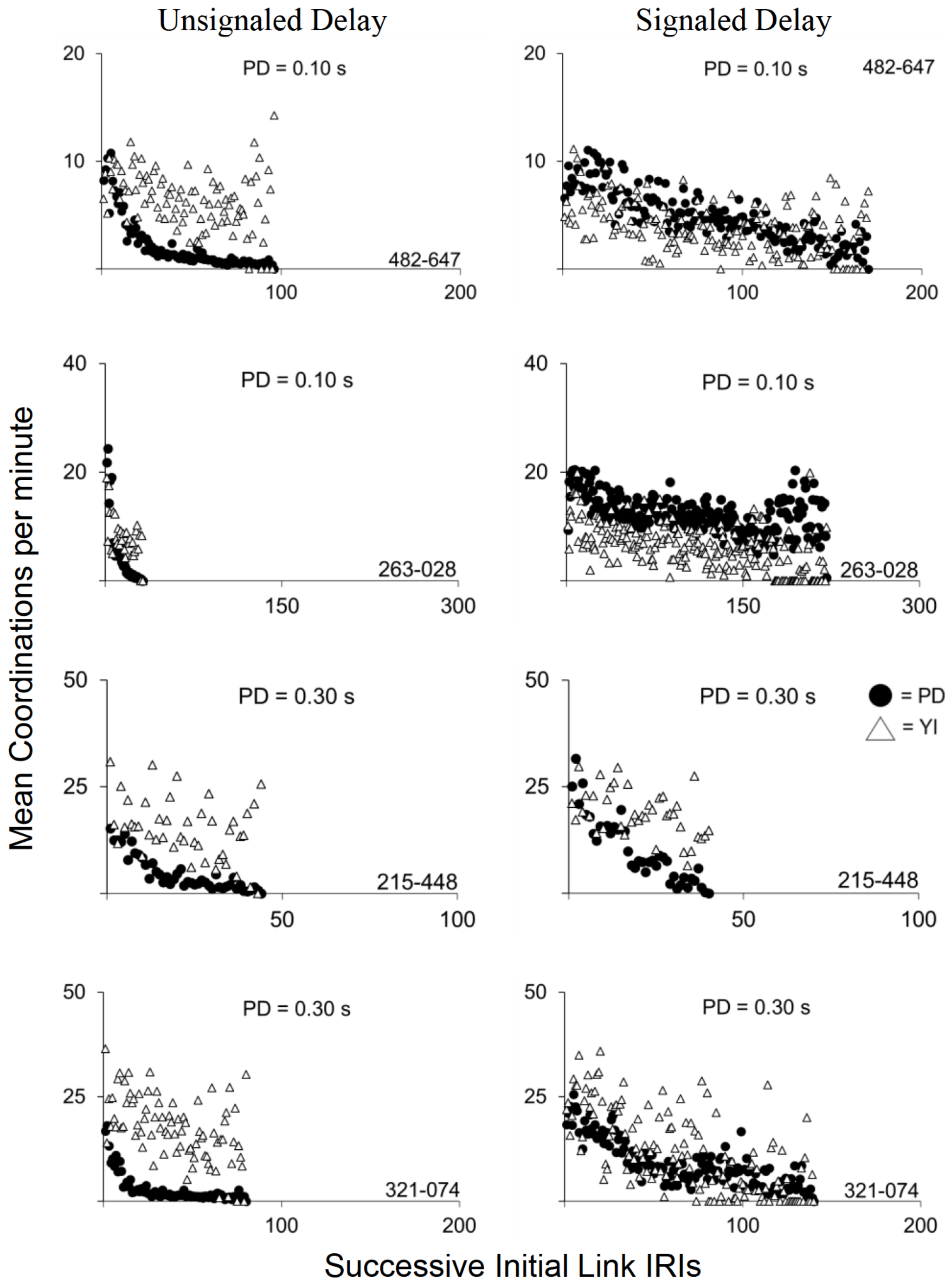

Figure S8: Coordination rates during the initial-link schedule under unsignaled and signaled delays of coordination-dependent reinforcement and their corresponding yoked-immediate reinforcement Experiment 2. Closed circles represent delayed-reinforcement conditions and open circles represent yoked-immediate-reinforcement conditions. Data points representing later IRIs were computed from fewer observations because fewer sessions reached those intervals.

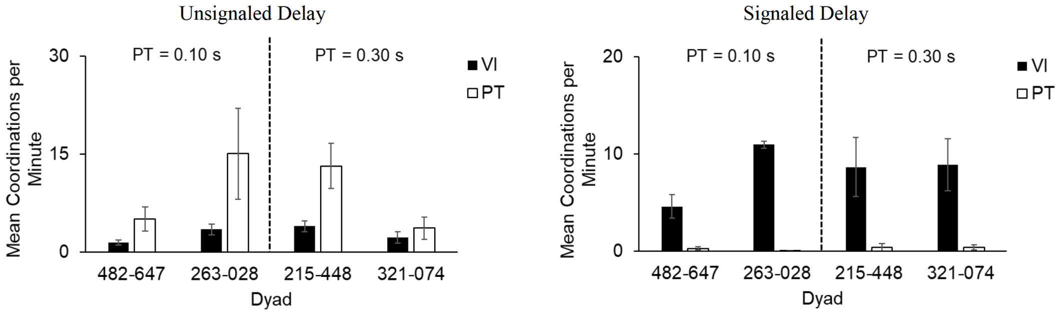

Figure S9: Mean coordination rates during the VI and PT links of unsignaled and signaled delays of coordination-dependent reinforcement Experiment 2. Filled bars represent coordination rates during the VI link and open bars represent coordination rates during the PT link. Panels on the left depict unsignaled delays, whereas panels on the right depict signaled delays. Dyads exposed to 0.10-s delay increments are shown to the left of the vertical dashed line, whereas dyads exposed to 0.30-s delay increments are shown to the right of the vertical dashed line. Error bars represent standard deviations computed from the last six sessions of each component.

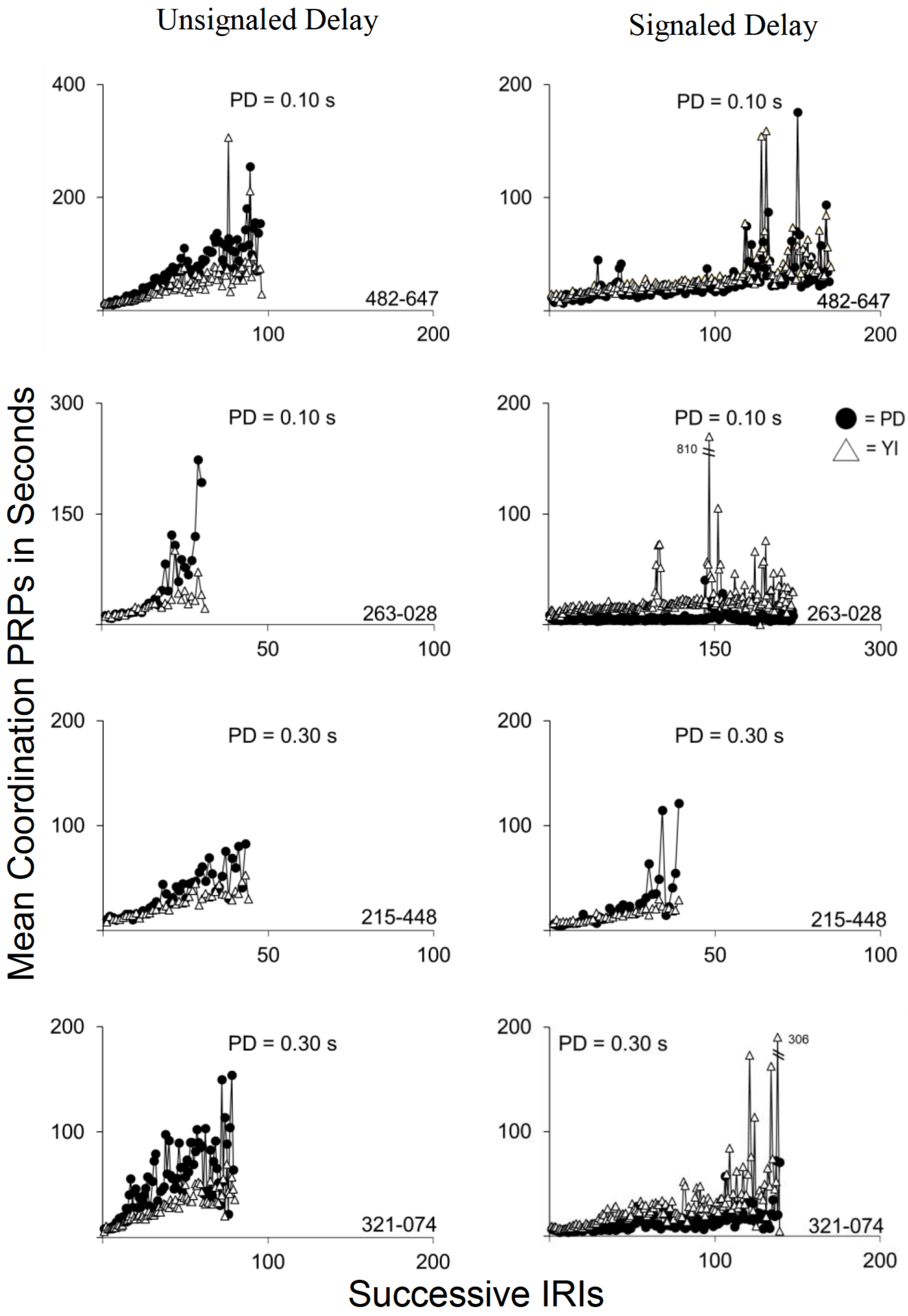

Figure S10: Mean coordination postreinforcement pauses (PRPs) across successive interreinforcer intervals (IRIs) under unsignaled and signaled delays of coordination-dependent reinforcement and their corresponding yoked-immediate reinforcement in Experiment 2. Closed circles represent delayed-reinforcement conditions and open triangles represent yoked-immediate-reinforcement conditions. Data points representing later IRIs were computed from fewer observations because fewer sessions reached those intervals.

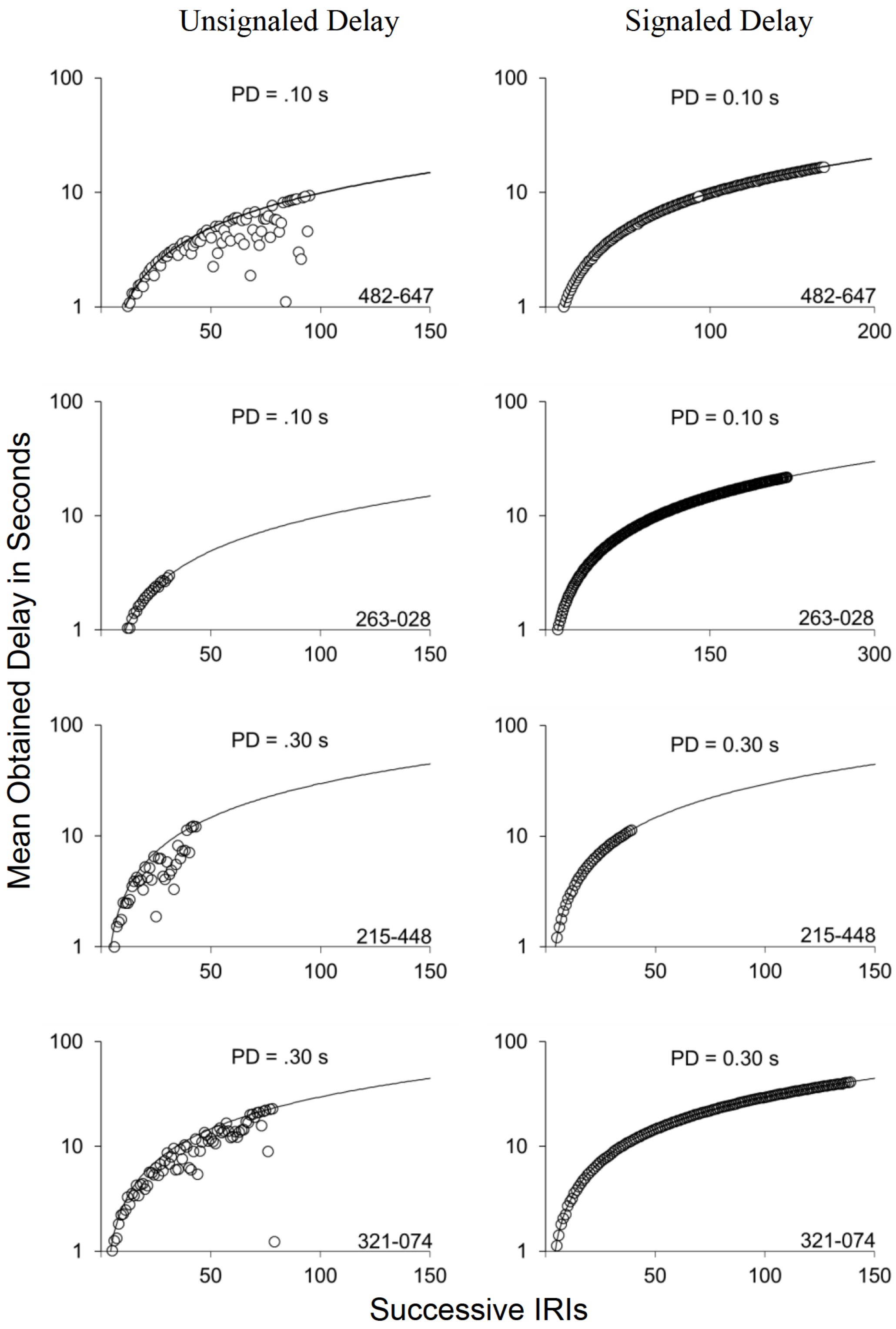

Figure S11: Mean obtained delays across successive interreinforcer intervals (IRIs) under unsignaled and signaled delays of coordination-dependent reinforcement in Experiment 2. Obtained delays are shown as open circles and programmed delays are shown as solid lines. The y-axis is plotted on a logarithmic scale. Data points representing later IRIs were computed from fewer observations because fewer sessions reached those intervals.

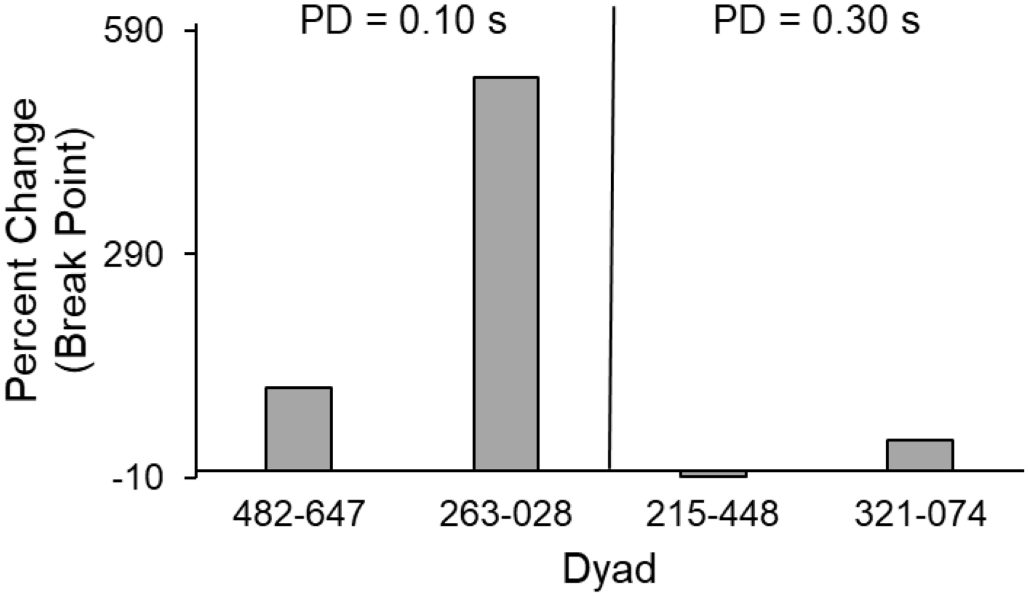

Figure S12: Percent change in median break points during signaled relative to unsignaled delays of coordination-dependent reinforcement for each dyad in Experiment 2. Percent change was calculated by subtracting the median break point obtained during the unsignaled delay component from the median break point obtained during the signaled delay component, dividing the difference by the unsignaled value, and multiplying by 100. Percent change values were computed from the last six sessions of each component. The solid vertical line separates dyads exposed to 0.10-s and 0.30-s delay increments.

Follow-Up Analysis for Dyad 215–448

These supplementary analyses were conducted for Dyad 215–448 in a follow-up condition. In this condition, only signaled and unsignaled coordination-dependent delayed reinforcement were compared across 12 sessions, and yoked-immediate-reinforcement conditions were not implemented. In addition, the progressive-delay increment was 0.50 s rather than 0.30 s as used in Experiment 2. These data are presented for descriptive purposes and should be interpreted cautiously.

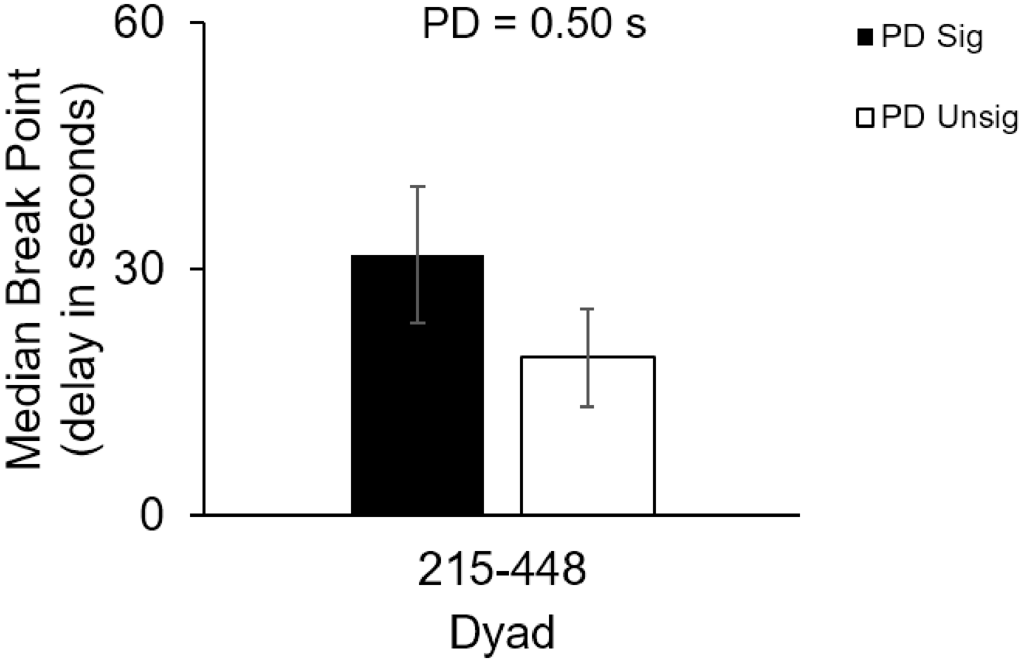

Figure S13: Median break points during signaled (blackout) and unsignaled delays of coordination-dependent reinforcement for Dyad 215–448 in a follow-up condition. Black bars represent signaled delays and white bars represent unsignaled delays. Error bars represent interquartile ranges computed from the last six sessions of each condition.

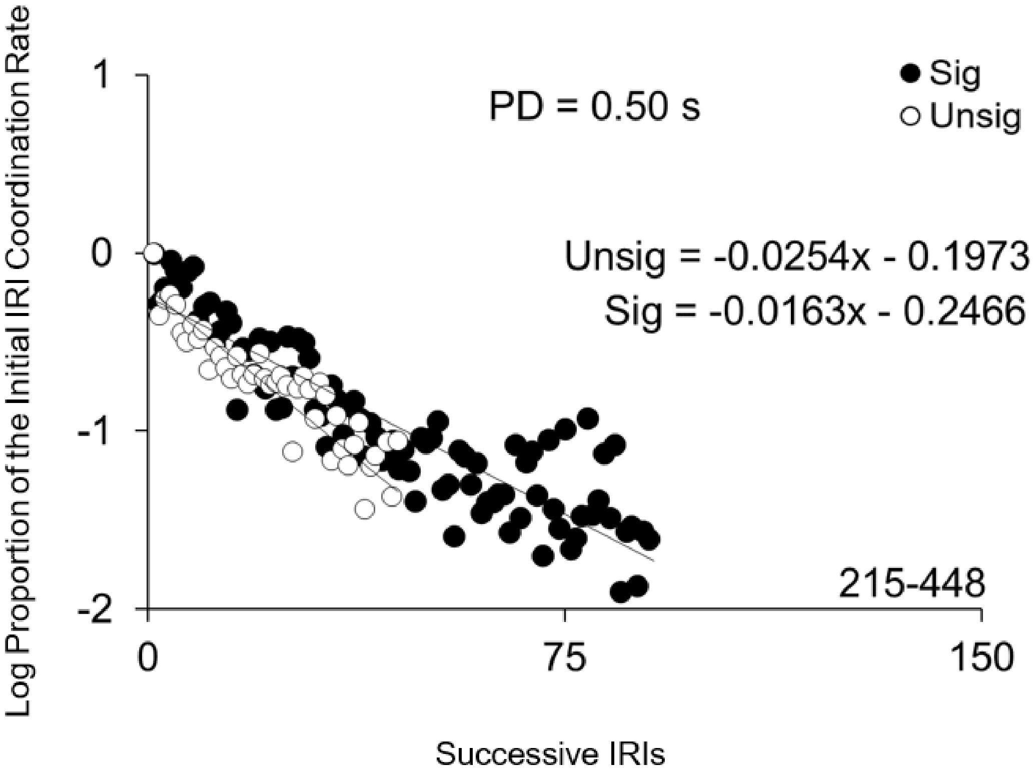

Figure S14: Log proportion of the initial interreinforcer interval (IRI) coordination rate across successive IRIs for Dyad 215–448 during signaled (blackout) and unsignaled delays of coordination-dependent reinforcement in a follow-up condition. Log proportions were calculated by dividing the coordination rate in each successive IRI by the coordination rate in the initial IRI and then transforming the resulting proportion using the logarithm function. Closed circles represent signaled delays and open circles represent unsignaled delays. Data points representing later IRIs were computed from fewer observations because fewer sessions reached those intervals.
